# A GC-MS/Single-Cell Method to Evaluate Membrane Transporter Substrate Specificity and Signaling

**DOI:** 10.3389/fmolb.2021.646574

**Published:** 2021-04-13

**Authors:** Stephen J. Fairweather, Shoko Okada, Gregory Gauthier-Coles, Kiran Javed, Angelika Bröer, Stefan Bröer

**Affiliations:** ^1^ Research School of Biology, Australian National University, Canberra, ACT, Australia; ^2^ Research School of Chemistry, Australian National University, Canberra, ACT, Australia; ^3^ Commonwealth Scientific and Industrial Research Institute (CSIRO) Land and Water, Canberra, ACT, Australia

**Keywords:** mTORC1 signaling, SNAT2, slc38a2, GC-MS, metabolomics, *Xenopus laevis* oocytes, amino acid transporters, amino acid signaling

## Abstract

Amino acid transporters play a vital role in metabolism and nutrient signaling pathways. Typically, transport activity is investigated using single substrates and competing amounts of other amino acids. We used GC-MS and LC-MS for metabolic screening of *Xenopus laevis* oocytes expressing various human amino acid transporters incubated in complex media to establish their comprehensive substrate profiles. For most transporters, amino acid selectivity matched reported substrate profiles. However, we could not detect substantial accumulation of cationic amino acids by SNAT4 and ATB^0,+^ in contrast to previous reports. In addition, comparative substrate profiles of two related sodium neutral amino acid transporters known as SNAT1 and SNAT2, revealed the latter as a significant leucine accumulator. As a consequence, SNAT2, but not SNAT1, was shown to be an effective activator of the eukaryotic cellular growth regulator mTORC1. We propose, that metabolomic profiling of membrane transporters in *Xe* *nopus laevis* oocytes can be used to test their substrate specificity and role in intracellular signaling pathways.

## Introduction

Mass spectrometry (MS) is a sensitive, high-throughput and relatively inexpensive technology for the identification of small metabolites in complex chemical matrices. With the standardization of chemical libraries and chemical fragmentation conditions, gas chromatography-mass spectrometry (GC-MS) and liquid chromatography-mass spectrometry (LC-MS) have become ‘gold standards’ for metabolomics research (Fiehn, 2016), advancing our understanding of biological metabolism [reviewed in (Sarker and Nahar, 2012; Hübschmann, 2015; Lu et al., 2017)]. Despite widespread use, MS has rarely been utilized to study the dynamic flux of primary metabolites across plasma membranes, a process mediated by membrane transporters. Membrane transporters are proteins embedded in and/or spanning a membrane bilayer and are grouped by genetic sequence similarity within the Solute Carrier (SLC) superfamily (Kandasamy et al., 2018). Mechanistically, they can be classified as uniporters, symporters and exchangers (antiporters), utilizing electrochemical gradients to drive substrate transport and can consist of single or multiple protein subunits of the same or different genetic origin (Fairweather et al., 2020). Although GC-MS and LC-MS have been sporadically used to measure transporter substrate specificity and activity (Grundemann et al., 2005; Abplanalp et al., 2013; Ebert et al., 2017) there has been little investigation of their potential utility in the study of the membrane flux of complex, physiologically relevant mixtures.

Single substrate techniques have been overwhelmingly used to understand transporter function [reviewed in (Broer, 2010; Grewer et al., 2013; Jani and Krajcsi, 2014; Fitzgerald et al., 2017; Broer and Fairweather, 2018)]. A large majority of known human transporters are capable of translocating multiple substrates across membranes and function in complex biological matrices. For instance, the amino acid exchanger LAT1-4F2hc (slc7a5-slc3a2) has over 50 known substrates to date (Uchino et al., 2002; Li and Whorton, 2005; Su et al., 2005; Wongthai et al., 2015; Zur et al., 2016; Ebert et al., 2017; Chien et al., 2018). Metabolomics utilizing GC- and/or LC-MS can expand our understanding of membrane transporters and related cellular phenotypes.

A widely used model for studying transport physiology are the oocytes of the female South-African clawed frog *Xenopus laevis* (Broer, 2010). This large single-cell system lends itself to measuring amino acid flux and metabolism as endogenous concentrations of most amino acids are low (Taylor and Smith, 1987; Meier et al., 2002). In addition, only two endogenous amino acid transport systems are detectable in oocytes, both expressed at much lower levels than heterologously expressed transporters (Taslimifar et al., 2017). Metabolic analysis using GC-MS has been performed once on *X. laevis* oocytes, however the aim was to conduct high sensitivity metabolomics from minimal sample volumes (Koek et al., 2010). Xenopus oocytes have also been utilized to reconstitute conserved multicellular signaling pathways by introducing a minimal number of exogenous membrane components (Vera and Rosen, 1990).

One potentially useful application of combining *X. laevis* transporter expression with metabolomic analyses would be to correlate intracellular amino acid levels with the status of intracellular signaling pathways such as mTOR and GCN2. The mTORC1 complex monitors cytosolic and lysosomal amino acid levels using indicative amino acids such as arginine, leucine (Hara et al., 1998; Christie et al., 2002; Beugnet et al., 2003; Nicklin et al., 2009; Jewell et al., 2015; Yoon et al., 2016; Zheng et al., 2016; Wyant et al., 2017; Lee et al., 2018), methionine (Gu et al., 2017), serine (Fan et al., 2016) and tryptophan (Christie et al., 2002). Both metabolic synthesis and plasma membrane uptake of glutamine have also been implicated in mTORC1 activation (Tan et al., 2017).

Multiple transporters have been advanced as direct or indirect activators of the mTORC1 pathway including SNAT2 (slc38a2) (Evans et al., 2007; Hyde et al., 2007; Evans et al., 2008), SNAT9 (slc38a9) (Rebsamen et al., 2015; Wang et al., 2015), LAT1-4F2hc (slc7a5-slc3a2) (Fuchs et al., 2007; Nicklin et al., 2009), ASCT2 (slc1a5) (Fuchs et al., 2007; Nicklin et al., 2009), PAT1 (slc36a1) (Heublein et al., 2010; Zoncu et al., 2011; Wu et al., 2016; Zhao et al., 2019) and PAT4 (slc36a4) (Heublein et al., 2010; Fan et al., 2016; Zheng et al., 2016). A number of studies have now firmly established that SNAT9 is a lysosomal arginine sensor for mTORC1 (Jung et al., 2015; Rebsamen et al., 2015; Wang et al., 2015; Rebsamen and Superti-Furga, 2016; Wyant et al., 2017) by direct interaction of the transport N-terminal with the Rag GTPase-Ragulator-FLCN:FNIP2 complex upon arginine sensing (Fromm et al., 2020; Lei et al., 2020). Another widely proposed mechanism is glutamine accumulation by ASCT2, followed by its exchange for leucine import via LAT1-4F2hc (Fuchs et al., 2007; Nicklin et al., 2009). However, ASCT2 knock-out studies in several cell lines have failed to observe a reduction of mTORC1 activity (Broer et al., 2000; Bode et al., 2002; Broer et al., 2016). SNAT2 and Golgi localized PAT4 have been suggested as mTORC1 activators by acting as membrane ‘transceptors’, where extracellular binding of substrate and allosterically-mediated signal transduction causes activation of mTORC1 (Goberdhan et al., 2005; Pinilla et al., 2011; Fan et al., 2016). For leucine, however, the transceptor mechanism appears unlikely as leucine-mediated mTORC1 activation has been shown to require an intracellular leucine increase (Christie et al., 2002; Beugnet et al., 2003).

In this study we combine the advantages of *X. laevis* oocytes in studying membrane transporter physiology with metabolomics to study membrane transporters in complex biochemical matrices. We show that single cell/metabolomics can be used to identify the comprehensive substrate profile of numerous amino acid transporters of various mechanisms in a time-efficient, robust manner using minimal sample preparation. As a proof-of-concept we identify the small neutral amino acid transporter SNAT2 as a direct and specific activator of mTORC1.

## Materials and Methods

### *Xenopus laevis* Surgery and Oocyte Preparation

Female *Xenopus laevis* frogs were anaesthetised by submersion for 30 min in 3-Aminobenzoic acid ethyl ester (1.5 g/L) (Sigma-Aldrich, St Louis, MO, United States). Anesthetized frogs were placed on ice to slow blood flow and surgery was conducted as previously described (Broer, 2010; Parker et al., 2019). Pieces of ovary were transferred into ${\text{OR}}^{2-}$ buffer (82.5 mM NaCl, 2.5 mM KCl, 1 mM MgCl_2_, 1 mM Na_2_HPO_4_, 5 mM Hepes-NaOH, pH 7.8) in a fresh Petri dish and cut into small clumps of ∼15 to 30 oocytes and were digested in 1.5 mg/ml collagenase D and 0.1 mg/ml collagenase A (Sigma-Aldrich) dissolved in ${\text{OR}}^{2-}$ (pH 7.8) buffer for 2−4 h at 28°C. Following digestion, oocytes were washed and examined for the extent of defolliculation and individual separation. Defolliculated oocytes were maintained in OR^2+^ buffer (OR^2−^ supplemented with 1.5 mM CaCl_2_ and 50 μg/ml gentamycin, pH 7.8) at 16−18°C. Maintenance of animals and preparation of oocytes was approved by the Australian National University animal ethics review board (ANU Protocol A2017/36).

### Transporter Expression in Oocytes

Transporter and other gene constructs were all subcloned into the pGem-He-Juel (pGHJ) expression vector as previously described (Supplementary Table S1). Constructs were linearized using *SalI* or *NotI* restriction endonucleases (NEB, Ipswich, MA, United States) and mRNA was synthesized using *in vitro* transcription with either T7 or SP6 mMessage mMachine kits (Ambion, Austin, TX, United States). After purification using phenol-chloroform extraction and precipitation, cRNA was quantified using a Nanodrop spectrophotometer and adjusted to 1 mg/ml (Thermo Fisher Scientific, Scoresby, VIC, Australia) (OD_260_/OD_280_) (Broer, 2003; Kowalczuk et al., 2008). Micro-injection of cRNA into oocytes was performed using a Micro4™ micro-syringe pump controller and A203XVY nanoliter injector (World Precision Instruments). The injection amount of all cRNAs was optimized previously or for this study (Broer et al., 2000; Broer et al., 2001; Bohmer et al., 2005; Kowalczuk et al., 2008; Fairweather et al., 2012). Oocytes were used 2–4 days post-injection unless otherwise indicated and as previously optimized for specific transporters. Oocytes were maintained in OR^2+^ (pH 7.8) buffer.

### Oocyte Flux Experiments

Flux experiments in oocytes have been detailed previously (Broer, 2010; Fairweather et al., 2015). Briefly, batches of 7–10 oocytes were incubated in 1  ×  ND96 oocyte assay buffer (96 mM NaCl, 2 mM KCl, 1 mM MgCl_2_, 1.8 mM CaCl_2_, 5 mM HEPES-NaOH pH 7.4) spiked with an unlabelled amino acid and 1.0 μCi/ml of L-[^14^C]- or L-[^3^H]-labelled amino acid at a final concentration of 100 μM in order to quantify the uni-directional flux. Oocytes were incubated with radiolabeled substrate for 20–30 min before the reaction was quenched by washing four times with ice-cold ND96 followed by transfer into a 96-well plate (PerkinElmer, Waltham, Massachusetts, United States). Oocytes were then lyzed using 10% (w/v) SDS and mixed with 5 × volume of scintillation fluid for radioactive counting in a MicroBeta^2^® 2450 96-well Microplate counter (PerkinElmer).

A modified formulation of Leibovitz’s L-15 cell culture medium was developed for oocyte incubation prior to GC-MS or LC-MS analysis. To this end, L-15 was supplemented with 20% (v/v) Fetal Calf Serum (FCS, Gibco), 20 mM HEPES and four amino acids absent from the standard formulation (see Supplementary Table S2). To adjust for osmolarity, the medium was diluted with 14% (v/v) MilliQ H_2_O. For metabolomics experiments, 12 oocytes were washed three times and then incubated in 35 mm cell culture dishes with 2 ml of modified L-15 for the indicated period of times. The number of oocytes used was optimized by balancing the requirement to detect all possible substrates for each transporter, even low affinity ones, with the need to keep large metabolite peaks under GC-Q detection threshold and to minimize the increase in endogenous oocyte metabolites such as TCA cycle intermediates and anionic amino acids. For sample extraction, oocytes were transferred to 1.5 ml Eppendorf tubes and washed four times with 1 ml of ice-cold MilliQ H_2_O before sample extraction for MS analysis (see below).

To analyze amino acid exchanger activity, oocytes were preloaded with either 10 mM of unlabelled substrate or unlabelled substrate supplemented with [^14^C]-labelled amino acid at 16°C for 24 h. All substrates were dissolved in ND96 oocyte assay buffer. Once pre-loading was complete oocytes were washed four times with 1 ml of ice-cold MilliQ H_2_O, or washed three times in modified L-15 depending on the experimental condition. For experiments where mTORC1 substrates were detected, oocytes were incubated with various media as indicated in figure legends, specifically either the L-15 based media, Leu alone at 1 mM or a 20 canonical amino acid mix with each amino acid at 1 mM concentration.

### Oocyte Metabolite Extraction and GC-MS Analysis

Preparation of all oocyte samples for GC-MS analysis was conducted following an established protocol for untargeted metabolomics (Carroll et al., 2010; Fiehn, 2016; Javed et al., 2018). Metabolites from oocyte samples were extracted using a 3:1:1 ratio of CH_3_OH:CHCl_3_:H_2_O (v/v) mixture. Ribitol (100 ng/μl) was added to each mixture as an internal standard. Samples were vortexed vigorously to lyse and homogenize oocytes and then centrifuged at 16,000 × g for 5 min at 4°C. The supernatant was transferred to a new tube and ½ volume equivalent of MilliQ H_2_O (0.2 μm-filtered) was added to extract hydrophilic metabolites. The samples were centrifuged again at 16,000 × g for 5 min at 4°C. The upper aqueous phase was transferred to a new tube and stored for up to 1 week at −80°C for later use. For immediate GC-MS analysis, 200 μl of the extract was transferred into 12 × 32 mm amber crimp top vials with 300 μl capacity inserts (5188-6594, Agilent Technologies, Palo Alto, CA, United States). The samples were dessicated using a miVac DUO concentrator (SP Scientific, Ipswich, United Kingdom) for 4 h and then sealed using 11 mm PTFE/rubber septa crimp caps (Agilent Technologies).

A single quadrupole GC/MSD instrument was used in this study, with the GC (Agilent 7890A) coupled to a single quadrupole mass spectrometer (Agilent 5975C). The GC was equipped with a J&W VF-5 ms column (30 m × 0.25 mm; 0.25 µm), connected to a 10 m EZ-Guard column (Agilent Technologies, Palo Alto, CA, United States). Two derivatization steps (methoximation and trimethylsilylation) and sample injection were performed by a robotic Gerstel MPS2 multipurpose auto-sampler and injector (GERSTEL GmbH and Co. KG, Mülheim an der Ruhr, Germany). To this end the dried extracts were first incubated with 10 µl of anhydrous pyridine (Sigma-Aldrich) containing 20 mg/ml methoxyamine hydrochloride (Sigma-Aldrich) at 37°C for 90 min. Subsequently, 15 µl of N-methyl-N-(trimethylsilyl) trifluoroacetamide (MSTFA; Sigma-Aldrich) was added at 37°C for 30 min to ensure maximal derivatization. For Kovats non-isothermal retention index (RI) matching, n-alkanes (5 µl of 29 mg/L, C12, C15, C19, C22, C28, C33, C36, Sigma-Aldrich) were also added to the derivatized samples and incubated for 1 min at 37°C, followed by injection onto the column. All samples were run in splitless injection mode, with 1 μl injected at a rate of 50 μl/sec. The inlet temperature was 230°C and helium (He_2_) was used as a carrier gas at a flowrate of 1 ml/min. The GC column oven was held at an initial temperature of 70°C for 1 min before increasing to 325°C at a ramp rate of 15°C/min. The solvent delay for the 15°C/min ramp was 6.2 min. Once the oven reached 325°C it was held for 3 min, making a total run time of 21 min/sample. The electron impact (EI) ion source and quadrupole were kept at 250 and 150°C, respectively. The filament current was set at 70 eV. The auxiliary transfer line was at 260°C and the quadrupole mass analyser was operated in full MS scan acquisition mode from 40 to 600 m/z using a scan rate of 3.6 Hz. Quality control (QC) samples were prepared by pooling aliquots from all sample extracts, dried down and derivatized as per oocyte extracts. Derivatized QC samples were injected at the start of the run and also after every 8 samples until the end of each run. Blank samples of 200 μl 1:1 CH_3_OH:H_2_O were run at the start and end of each batch to monitor column contaminants in addition to washing with 3 × injections of 1 μl methanol before and after the running of each batch. All batches included a standard mix containing 20 canonical amino acids plus L-ornithine that was dried down, derivatized and analyzed at the beginning, mid-run and at the end of each batch. Amino acids with good response factors were added at 1 µg/amino acid, those with poorer response factors (L-Arg, L-Ala, L-His, L-Trp, L-Cys) were added at 10 µg/amino acid.

For the quantification of endogenous *X. laevis* oocyte amino acids calibration curves from ultrapure amino acid standards (Sigma) were generated. The linear range of each amino acid was determined first and as a result they were run as grouped standards based on their comparative response factors (R_f_), either as high, medium, or low R_f_ amino acids. Standards were interspersed throughout runs and six data points used per amino acid to generate linear nmol vs peak height plots. Concentrations of endogenous amino acids were calculated and converted to pmol/oocyte based on a previously determined water-accessible volume of stage 5–6 oocytes (Stegen et al., 2000) and compared with previously determined values using HPLC (Table 1) (Taylor and Smith, 1987; Meier et al., 2002). We further confirmed that trimethylsiyl (TMS) derivatization of detected metabolites was consistent and produced a predominately single TMS-derivative of potential substrates (Supplementary Table S3). The proportional TMS-derivatisation of all 20 proteinogenic amino acids and some of their GC-MS by-products demonstrated that, besides aspartic acid and serine, all were detected by a single TMS-variant at greater than 80% of the total signal. All raw GC-MS raw data sets are available on the MetaboLights repository (www.ebi.ac.uk/metabolights/MTBLS2476) (Haug et al., 2020).

**TABLE 1:** Free endogenous amino acid pools in stage 5 and 6 *Xenopus laevis* oocytes.

GC-MS^a^				HPLC
Amino acid	EIC (m/z)	Concentration (μM)	pmol/oocyte (mean ± S.E.M)	Range^b^ (pmol/oocyte)
Valine	144	499 ± 121	182 ± 55	40–195
Leucine	158	77 ± 25	28 ± 9	20–44
Isoleucine	158	110 ± 36	40 ± 13	16–34
Proline	142	310 ± 110	113 ± 40	30–83
Glycine	174	64 ± 9	23 ± 3	22–92
Serine	204	290 ± 38	106 ± 14	112–263
Alanine	188	222 ± 33	81 ± 12	32–94
Threonine	218	96 ± 33	35 ± 12	16–106
Aspartic acid	232	2349 ± 1050	857 ± 383	409–2200^d^
Methionine	176	124 ± 52	45 ± 19	6–27
Cysteine	220	58 ± 16	21 ± 6	9–21
Glutamic acid	246	1869 ± 669	682 ± 244	793–2000^c^
Phenylalanine	216	93 ± 38	34 ± 14	10–33
Asparagine	231	650 ± 214	237 ± 78	—
Glutamine	156	1455 ± 104	531 ± 38	—
Arginine	157	288 ± 105	105 ± 38	51–188
Lysine	156	85 ± 44	31 ± 16	25–195
Histidine	154	121 ± 47	44 ± 17	28–236
Tyrosine	280	493 ± 80	180 ± 29	27–92
Tryptophan	202	33 ± 11	12 ± 4	—

^a^ Concentrations calculated for oocytes assuming an intracellular water-accessible volume of 365 nL as previously reported (Stegen et al., 2000).

^b^ Concentrations taken from (Taylor and Smith, 1987; Meier et al., 2002) for HPLC measurements take into account the full range of reported values included means ± S.D.

^c^ Includes values for both glutamate and glutamine combined.

^d^ Includes values for both aspartate and asparagine combined.

### GC-MS Sample Data Analysis

For GC-MS data acquisition the Agilent MSD Chemstation software (version E.02) was used. The acquired MS data was converted into CDF format by Agilent MSD Chemstation software (Agilent Technologies). Ribitol was used as the internal standard spiked into all GC-MS samples to ensure consistency of oocyte sample extraction by measuring m/z peak height and integrated area in every sample. If the ribitol signal deviated by more than 10%, then the samples were discarded. The peaks associated with the internal standard (ribitol) and n-alkanes standards for R.I. calculation (n-alkanes) were removed from processed data sets unless otherwise shown. Any identified m/z peaks which increased over the time of a batch run independently of any other variable was removed. Any column contaminants as observed from sample blanks derivatized blank samples were also removed.

Peak extraction and quantification from total ion chromatograms (TICs) was conducted using the MetabolomeExpress online software (https://www.metabolome-express.org/) according to published protocols (Carroll et al., 2010; Javed et al., 2018). Briefly, for each GC-MS run the retention times of internal alkane standards were used to generate a R.I. calibration file for metabolite identification by MetabolomeExpress. Raw data files were loaded onto the MetabolomeExpress server and peaks detected using the following parameters (all units are arbitrary MS abundance units unless otherwise stated): slope threshold = 200, min. peak area = 1,000, min peak height = 500, min. peak purity factor = 2, min. scan threshold for peak width = 5. Peak detection was followed by peak identification using the following parameters: RI window = 4, MST centroid distance = ± 1R.I., min peak area = 1,000, MS Qualifier Ion Ratio Error Tolerance = 30%, min number of correct ratio MS qualifier ions = 2, max. average MS ratio error = 70%, min similarity product = 60%. The two libraries used for metabolite identification were the Pooled Tea. MSRI (Public MetabolomeExpress Library) and Golm Metabolome database (GMD, http://gmd.mpimp-golm.mpg.de/).

Extracted peak tables (EIC) were exported as. mzrtMATRIX files for further metabolite identification and analysis using additional software. Several criteria were used to unambiguously identify metabolites. Identification criterion 1 (Supplementary Table S4) utilized the GMD and automated identification in the AMDIS program (Hummel et al., 2010). For this MetabolomeExpress was used for peak deconvolution prior to identification by AMDIS, to overcome a previously highlighted flaw in AMDIS, where m/z peaks were not reported if not present in all samples (Lu et al., 2008; Behrends et al., 2011). This approach also assisted in helping to remove any false positive metabolite identification by AMDIS. Extracted peaks were verified (a) by correct R.I. (±3.0 R.I. units), and (b) the presence of four characteristic m/z peaks (with a score of ≥850 for overall metabolite similarity). For inclusion in the analysis, symmetry about the peak centroid, and a minimal abundance of ≥500 units of the AMDIS deconvoluted peak were required. Verification of the 20 canonical amino acids following criterion 1 combined with the use of standards represents identification criterion 2. EIC quantification was derived only from MetabolomeExpress as AMDIS does not use a common m/z ion fragments when quantifying the same metabolite across multiple samples (Aggio et al., 2011). If a metabolite could not be identified by criteria 1 or 2, we used automated and manual searches of the NIST library. Any metabolite identified with a reverse spectral (head to tail) match score of ≥700 and verified by the presence of the two most abundant m/z peaks was listed as identified by criterion 3 (Supplementary Table S4). Using these 3 criteria, all peaks were further verified manually using the GMD and the Human Metabolome database (HMDB, http://www.hmdb.ca/). Any manual identification or verification of EIC identity excluded the use of major TMS peaks at 73 and 147 m/z.

### Oocyte Sample Extraction and LC-MS Analysis

To corroborate data from GC-MS and radiolabeled amino acid uptake experiments, we used LC-MS (Supplementary Table S5). Samples were prepared from oocytes using the same extraction protocol as for GC-MS with the exception that ribitol was substituted with 2.4 μl [^13^C]labeled amino acids (MSK-A2-1.2 and CLM-1822-H-0.1; Cambridge Isotope Laboratories; pooled together at a concentration of 500 µM) as the internal standards. Dried extracts were dissolved in 10 mM ammonium acetate:acetonitrile (15:85 + 0.15% formic acid) for LC-MS analysis.

Amino acids for SNAT4 and ATB^0+^ flux assay (Figure 4A) were detected using an Orbitrap Q-Exactive Plus (Thermo Fisher) coupled to an UltiMate 3000 RS UHPLC system (Dionex) and separated by a 3 µm ZIC cHILIC 2.1 × 150 mm column (EMD Millipore). The mobile phase was composed of A (10 mM ammonium acetate +0.15% formic acid) and B (acetonitrile + 0.15% formic acid) and was delivered at a flow rate of 0.4 ml/min with the following gradient elution: 15% A from 0 to 5 min, then a linear increase to 36% at 10 min, followed by an increase to 64% at 12.1 min where it was held until 17 min before returning to 15% at 17.1 min where it was maintained until the end of the run to re-equilibrate the column. The total run time for each sample was 21 min. Sample injection volume was 4 µl and the column oven temperature was kept at 35°C. Analytes were ionized in positive mode by heated electrospray ionization with an ion spray voltage of 3,500 V. Sheath gas flow was set to 50 units, auxiliary gas flow to 13 units and sweep gas flow to 3 units. Capillary temperature was 263°C and auxiliary gas heater temperature was 300°C. Analysis was performed in full scan mode (R = 35.000) and quantification was achieved using Xcalibur (Thermo Fisher) by comparing the area under the peak of each amino acid and their respective isotope-labelled internal standard. Data for cRNA injected oocyte samples was represented by fold changes over the un-injected oocyte controls incubated in matched conditions. Blanks were included throughout each sequence run to monitor analyte carry-over and external standards were included at the beginning and end of each sequence for quality-control purposes.

For the detection of uninjected oocyte metabolites (Supplementary Table S5), slight modifications were made to the chromatographic method. The gradient started with 80% mobile phase B (Acetonitrile; 0.1% v/v Formic acid) and 20% mobile phase A (10 mM ammonium formate; 0.1% v/v formic acid) at a flow rate of 300 μl/min, followed by a linear gradient to 20% mobile phase B over 18 min. A re-equilibration phase of 12 min using 80% mobile phase B was done with the same flow rate, making a total run time of 30 min. The column temperature and injection volume remain unchanged to the above method. The settings for the mass detection were also changed slightly with resolution set to 70,000 m/z range 60–900, AGC target set to 3 × 10^6^, sheath gas set to 40 units, auxiliary gas set to 10 units, sweep gas flow set to 2 units, capillary temperature set to 250°C, and spray voltage set to +3.5 kV. The MS/MS data was collected through data dependant top 5 scan mode using High-energy C-trap Dissociation (HCD) with resolution 17,500 m/z, AGC target set to 1 × 10^5^ and Normalized Collision Energy (NCE) 30%. A pooled sample of all extracts was used as a quality control (QC) sample to monitor signal reproducibility and stability of analytes. For LC-MS/MS data analysis of un-injected oocytes metabolites, the acquired raw metabolite data were converted into mzXML format and processed with the open-source software MS-DIAL (Tsugawa et al., 2015). The identification was done using publicly available MS/MS libraries by matching exact mass (MS tolerance 0.01 Da) and mass fragmentation pattern (MS tolerance 0.05 Da). Putative metabolite identifications were then confirmed by the use of standards. Raw peak height was used for the quantification of metabolites.

### Phylogenetic Identification and Analysis of *X. laevis* mTORC1 Substrates

*X. laevis* homologues of human mTORC1 pathway proteins were identified by using the human protein sequence to create a profile Hidden Markov Model (HMM) using the phmmer algorithm at the HMMER project (Potter et al., 2018) (Dates 12-2-2020 to 16-4-2020). For each mTORC1 pathway protein the HMM profile was searched against all reference proteomes with the following options: sequence e-value = 0.01, e-value hit = 0.03, open gap penalties = 0.02, extend gap penalties = 0.4, substitution rate matrix = BLOSUM62. Taxonomic filters were set to limit results to human and amphibians. Multiple sequence alignments for each protein were constructed using PROMALS3D (Pei and Grishin, 2007) and analyzed for conservation using BioEdit 7.2 followed by selection of the highest ranked *X. laevis* homologue. The homology of mTORC1 *X. laevis* and human proteins were verified using OrthoDB v10.1.

### Western Blotting on Oocytes of mTORC1 Substrates

Electrophoresis was performed using 4–12% Bis-Tris polyacrylamide NuPAGE gels (Thermo Fisher Scientific) in an XCell SureLock_Mini-Cell (Thermo Fisher Scientific) under reducing conditions according to standard procedures and using either MES or MOPS buffer. The SeeBlue Plus 2 pre-stained protein ladder (Thermo Fisher Scientific) was used to estimate the apparent molecular weight of proteins. Following SDS-PAGE, proteins were transferred onto nitrocellulose membranes (GE Healthcare) using the Mini Trans-Blot Electrophoretic Transfer Cell (Bio-Rad) according to standard protocols. Blots were blocked for 2 h at room temperature (or overnight at 4°C) in 50 ml of 10% (w/v) skim milk in PBS, pH 7.4, with 0.15% Tween 20 (PBS-T). After washing thrice in PBS-T for 10 min each, the blots were incubated with the primary antibody for 2 h or overnight in 5 ml of skim milk (2%, w/v) in PBS-T. The following primary antibodies and dilutions were used: anti-4E-BP1 (1:2,000) (catalogue #9644), anti-Phospho-4E-BP1 (Thr37/46) (1:2,000 (#2855), anti-S6 (1:2,000) (#2217), anti-Phospho-S6 (Ser240/244) (1:1,000) (#5364), anti-p70S6K (1:1,000) (#2708), anti-Phospho-p70S6K (Ser371) (1:1,000) (#9208) (all Cell Signaling Technology). All antibodies to human immunogens were raised in rabbit and were previously validated against mTORC1 targets (Broer et al., 2016). The HRP-conjugated anti-rabbit secondary IgG was used at 1:2,000 dilution for all primary antibodies (Cell Signaling Technology). Excess primary antibody was removed by washing thrice in PBS-T. Blots were incubated with 5 ml of diluted secondary antibody for 2 h. After washing thrice in PBS-T and a final rinse in PBS, immunoreactive bands were detected by enhanced chemiluminescence using Luminata Crescendo or Luminata Forte Western HRP Substrate (Millipore Merck). For reprobing, the same blots were incubated for 30 min at 70°C in 50 ml of stripping buffer (62.5 mM Tris-HCl, pH 6.8, 2% SDS, 100 mM 2-mercaptoethanol) and then alternate proteins detected by the same procedure as outlined. Western blots were quantified, where indicated, by densitometry analysis using the ImageJ 1.53e.

### Statistical and Data Analysis

In addition to Metabolomic software outlined above for metabolite peak extraction, deconvolution, and identification, the software package OriginPro 2020b was used for data analysis and graphics suite CoralDraw v7 and v22 for figure generation. All experimental groups were checked for both normality of the distribution using the Shapiro-Wilks test and homogeneity of the variance using Levene’s test. Metabolite levels of transporter-expressing oocytes were first normalized by subtracting un-injected oocytes signals for each metabolite incubated in the same media for the same time period. Paired *t*-tests were used to compare the significance of mean differences between the same metabolite across two experimental conditions. Calibration curves for quantification of endogenous amino acids were fitted to linear functions and adjusted *R* ^2^ values were computed. The adjusted *R* ^2^ values for all single amino acid linear regression were greater than or equal to 0.94.

## Results

### Validation of *X. laevis* Oocytes for Untargeted Metabolomics Profiling

**FIGURE 1:**
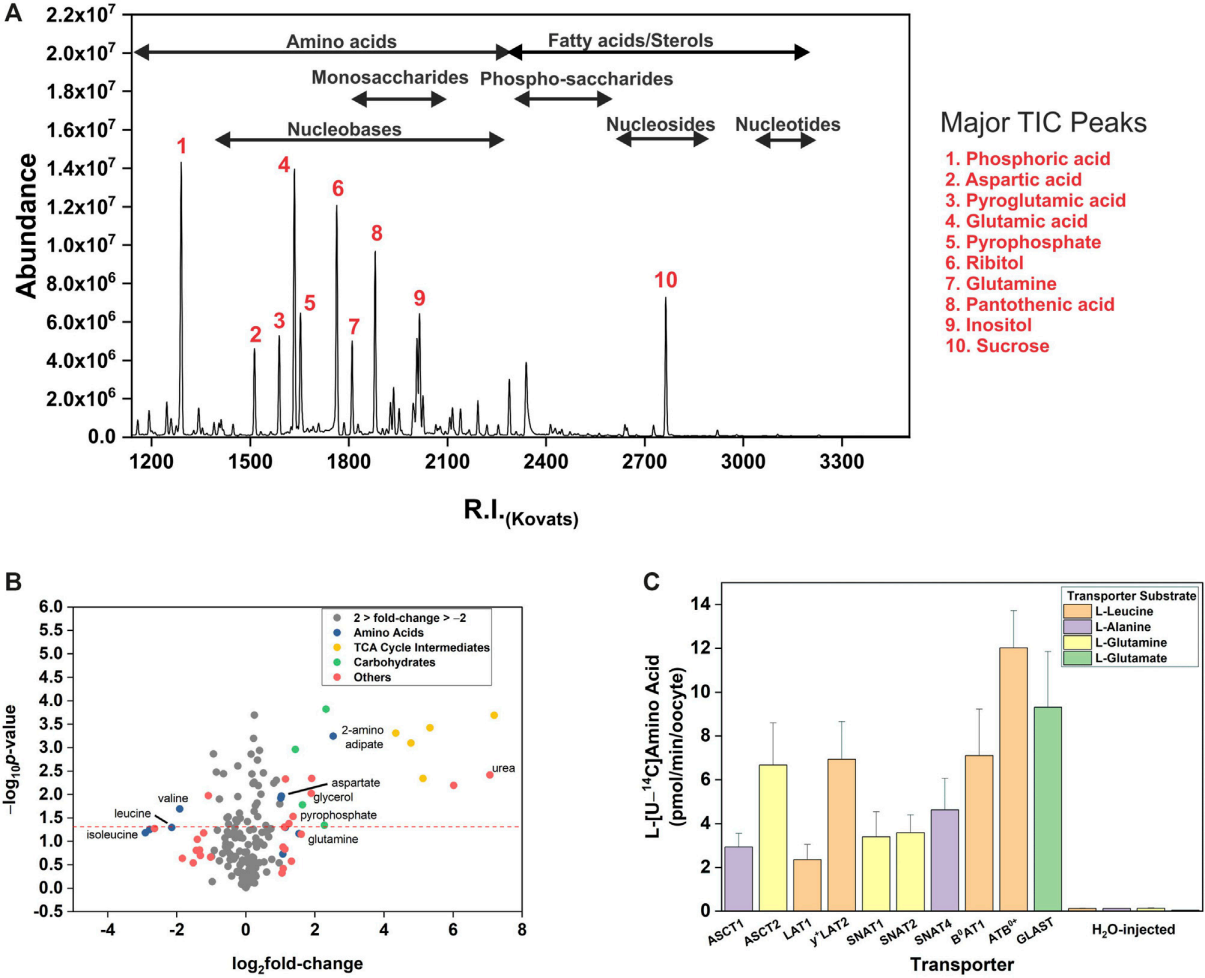
Metabolic profile of *Xenopus laevis* oocytes. **(A)** The total ion chromatogram (TIC) of South African clawed frog *Xenopus laevis* oocytes. The clusters of selected metabolite classes are indicated. Oocytes were analyzed after 4 days incubation in 1 × OR^2+^ buffer. A 3:1:1 methanol:chloroform:water extraction process was used followed by automated derivatisation/methoxymation. TIC acquisition was carried out on an Agilent 7890A gas chromatograph coupled to a 5975C single quadrupole mass spectrometer. This TIC is a representative of the un-injected oocytes utilized as controls throughout this study. Numbering and the legend indicate selected abundant metabolite peaks. **(B)** Un-injected oocytes were incubated in modified L-15 media for 4 h or remained in OR^2+^ followed by metabolite extraction and GC-MS analysis. Each data point in the volcano plot represents a single metabolite. The figures are a representative example of 3 repeats (*n* = 3 technical replicates comparing 12 L-15 incubated and 12 OR^2+^ incubated oocytes per replicate, *e* = 3). **(C)** Oocytes were injected with the 5–10 ng cRNA encoding transporters as listed. Transport activity was measured by 30 min incubation in 1  ×  ND96 (pH 7.4) assay buffer spiked with an unlabelled amino acid and 1.0 μCi/ml of L-[^14^C]- or L-[^3^H]-labelled amino acid to a final concentration of 100 μM. The reaction was quenched by washing four times with ice-cold 1  ×  ND96 (pH 7.4). Transport activity of H_2_O-injected oocytes is shown for comparison.

Using two metabolomic analysis software packages and manual verification we identified 141 metabolites with GC-MS (Supplementary Table S4). The distribution density of major metabolite classes showed that amino acids ranged between values of 1180.0–2218.3, while monosaccharides were clustered from RI 1743.0 to 2078.1 (Figure 1A). In addition, we observed sugar phosphates, nucleotides, sterols and lipids clustered between RI 1992.0 and 3191.8. We were also able to detect all TCA cycle intermediates, numerous vitamins and other enzyme co-factors (Supplementary Table S4). Abundant metabolites characteristic for stage 5/6 *X. laevis* oocytes were pantothenic acid (vitamin B_5_, RI 1984.3), sucrose (RI 2715.5), glutamic acid (RI 1616.1 and RI 1523.9 as pyroglutamic acid), and aspartic acid (RI 1421.1 for 2TMS derivative and 1508.0 for 3TMS derivative). After testing between six and 40 oocytes per experimental condition, the number required for robust and wide-ranging metabolite identification was established at twelve. Especially important was the ability to detect and identify all 20 proteinogenic amino acids and their main GC-MS by-products. As previously noted, substantial quantities of arginine are converted into ornithine, and of glutamate and glutamine into pyroglutamic acid (Leimer et al., 1977). However, all three amino acids were still detected chemically unaltered at lower abundance. We next established accurate endogenous concentrations for free AA in oocytes by generating calibration curves for all 20 proteinogenic AAs and quantifying them using LC-MS and GC-MS, with Table 1 showing GC-MS derived endogenous concentrations. As previously reported, all neutral and cationic AA were calculated to have endogenous intracellular oocyte concentrations below <500 μM with the exception of valine, asparagine and glutamine. Aspartate and glutamate were more abundant at ∼2 mM. These data are in the range of previous results (Taylor and Smith, 1987; Meier et al., 2002), except that glutamate was lower in our oocyte preparation, while isoleucine, proline, methionine and tyrosine were higher, although none beyond the range of previous values if standard deviations are taken into account.

Our LC-MS metabolomics method was able to identify 38 metabolites, of which 31 had also been identified by GC-MS (Supplementary Table S5). Some water-soluble metabolites such as biotin, betaine, choline, citrulline, maltose and phosphocreatine were detected by LC-MS but not by GC-MS. Some of these are not present in the GOLM metabolite database (http://gmd.mpimp-golm.mpg.de/), indicating that GC-MS detection of these metabolites might be difficult and that our metabolomics analysis of *X. laevis* oocytes was not fully comprehensive.

### Substrate Profiles of Membrane Transporters Measured by GC-MS

Oocytes from *X. laevis* have long been utilized for the comprehensive analysis of membrane transporters. To understand endogenous permeability, we wanted to observe if endogenous oocyte metabolites fluctuate during incubation in a complex substrate matrix (biomimetic L-15 medium). Due to the large volume, un-injected oocytes required greater than 3 h incubation for detection of the lowest response factor amino acid alanine (R.I. 1360). Transporter-injected oocytes also required >2 h incubation in order to detect changes of all substrate amino acids. As a result of this optimization, 4 h incubations were used for all subsequent transporter experiments. The majority of metabolites identified in un-injected oocytes showed a less than 2-fold (log_2_ = 1) increase or decrease after 4 h incubation (Figure 1B). Several TCA cycle intermediates, numerous monosaccharides, urea, pyrophosphate and glycerol were increased > 2-fold. Amino acids showed a divergent response with aspartate, glutamine, and 2-aminoadipate increasing >2-fold, while branched-chain amino acids (BCAA) valine, leucine and isoleucine decreased >2-fold. This analysis suggests that endogenous oocyte metabolism needs to be considered during long-term incubation. Prior to the measurement of transporter-mediated membrane flux using GC-MS, functional expression of all transporters was confirmed using radiolabeled substrates (Figure 1C).

**FIGURE 2:**
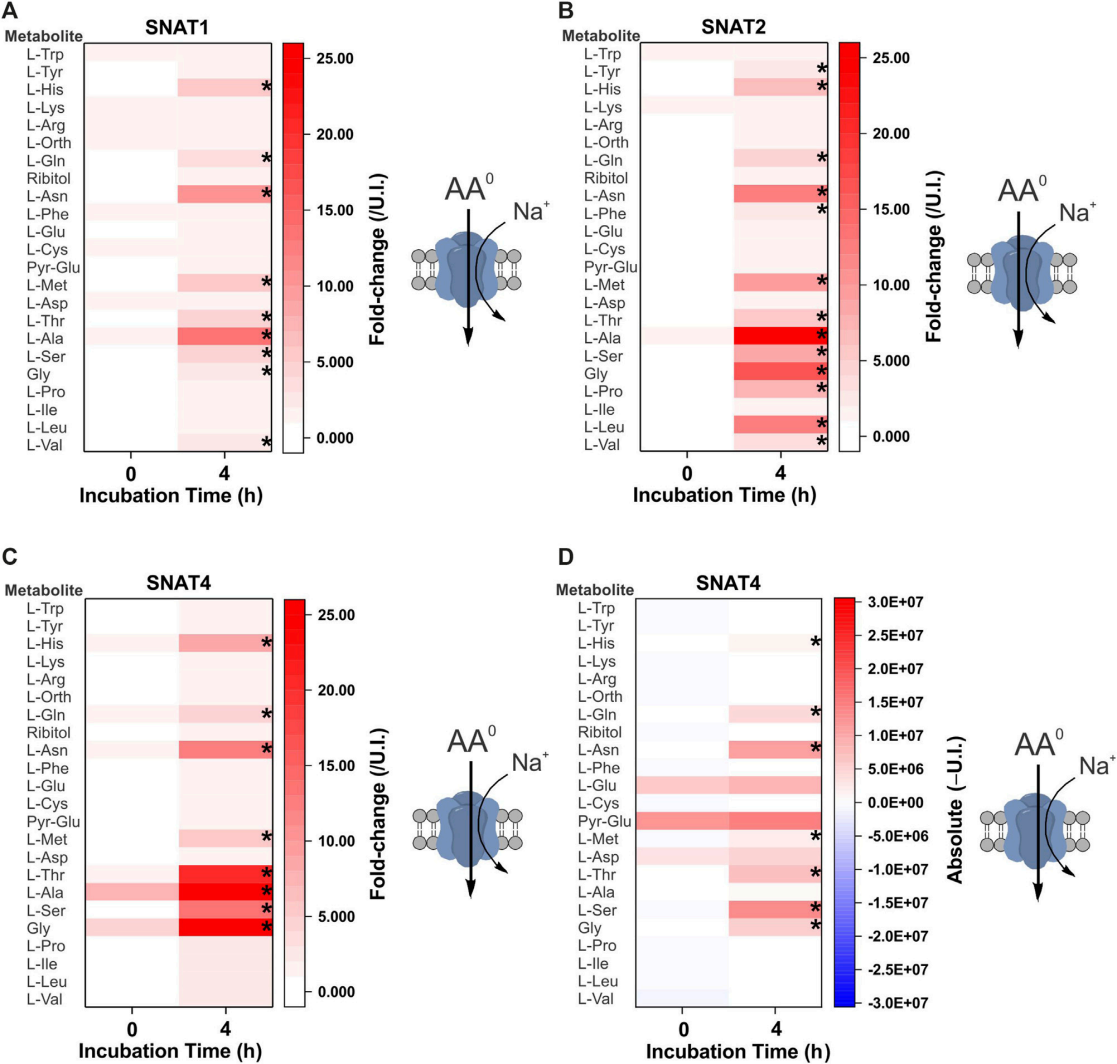
Comprehensive amino acid flux profile of human SNAT variants. Oocytes were injected with 10 ng of cRNA of SNAT1/2/4 transporters or remained un-injected (controls). After 4 days of incubation in OR^2+^ (pH 7.8), oocytes were incubated for 4 h in modified L-15 medium before metabolite extraction and analysis. Heat-maps show fold-change **(A–C)** or absolute peak difference **(D)** relative to un-injected oocytes. The published specificity and stoichiometry of transporters is shown as a cartoon to the right of each heat map. The fold-change **(A–C)** at each time point (0 and 4 h) was calculated by dividing the absolute signal for each amino acid by the corresponding signal from un-injected oocytes, thereby taking account of any natural fluctuations due to endogenous acquisition pathways during incubation. The absolute value heat-map **(D)** was generated by subtracting the signal of un-injected oocytes from the signal of transporter expressing oocytes at each time point (0 or 4 h). A more intense red for the same amino acid at 4 h indicates active accumulation. Pyro-glutamic acid (2-oxo-proline) and ornithine were included in all heat maps as they are known GC-MS derivatives of glutamate and arginine, respectively. The internal standard ribitol was also included in each heat map as a negative control to indicate any deviation from fold-change of 1 ± 0.1. * Indicates a difference in the means between the two time points at the *p* < 0.05 level using a paired *t*-test as calculated from the absolute mass spectrometer raw values. The figures are a representative example of 3 experiments with *n* = 12 transporter-expressing and un-injected oocytes per replicate.

The first cohort of membrane transporters tested were the sodium-neutral-amino-acid symporters of the SNAT (slc38) family, which are ubiquitously expressed and represent the major acquisition pathway for neutral amino acids in many healthy and malignant human cells (Broer and Broer, 2017; Broer and Fairweather, 2018) (Figure 2). Symporters are well-suited to the study of metabolite fluxes following complex matrix incubation as they are able to accumulate intracellular substrates. The GC-MS analysis broadly replicated previously reported substrate specificity with some exceptions. Notably, SNAT2 was able to accumulate a broader range of neutral amino acids than SNAT1, including proline, leucine, and to a smaller extent phenylalanine and tyrosine (Figures 2A,B). Both had been previously characterized as having similar specificity for small and polar neutral amino acids with lower affinity for hydrophobic neutral amino acids (Hatanaka et al., 2000; Wang et al., 2000). SNAT4 had a similar substrate spectrum as SNAT1, with a preference for smaller amino acids, such as glycine, alanine, serine, asparagine and threonine. In contrast to previous reports (Sugawara et al., 2000; Hatanaka et al., 2001), we could not find evidence for accumulation of cationic amino acids from a complex substrate matrix–only the amphiprotic histidine was accumulated (Figure 2C). Fold-change analysis can miss accumulation by SNAT4 of solutes that are naturally abundant in oocytes. As a result, we also analyzed absolute signal differences, but were still unable to detect significant increases of arginine or lysine in SNAT4-expressing oocytes (Figure 2D).

**FIGURE 3:**
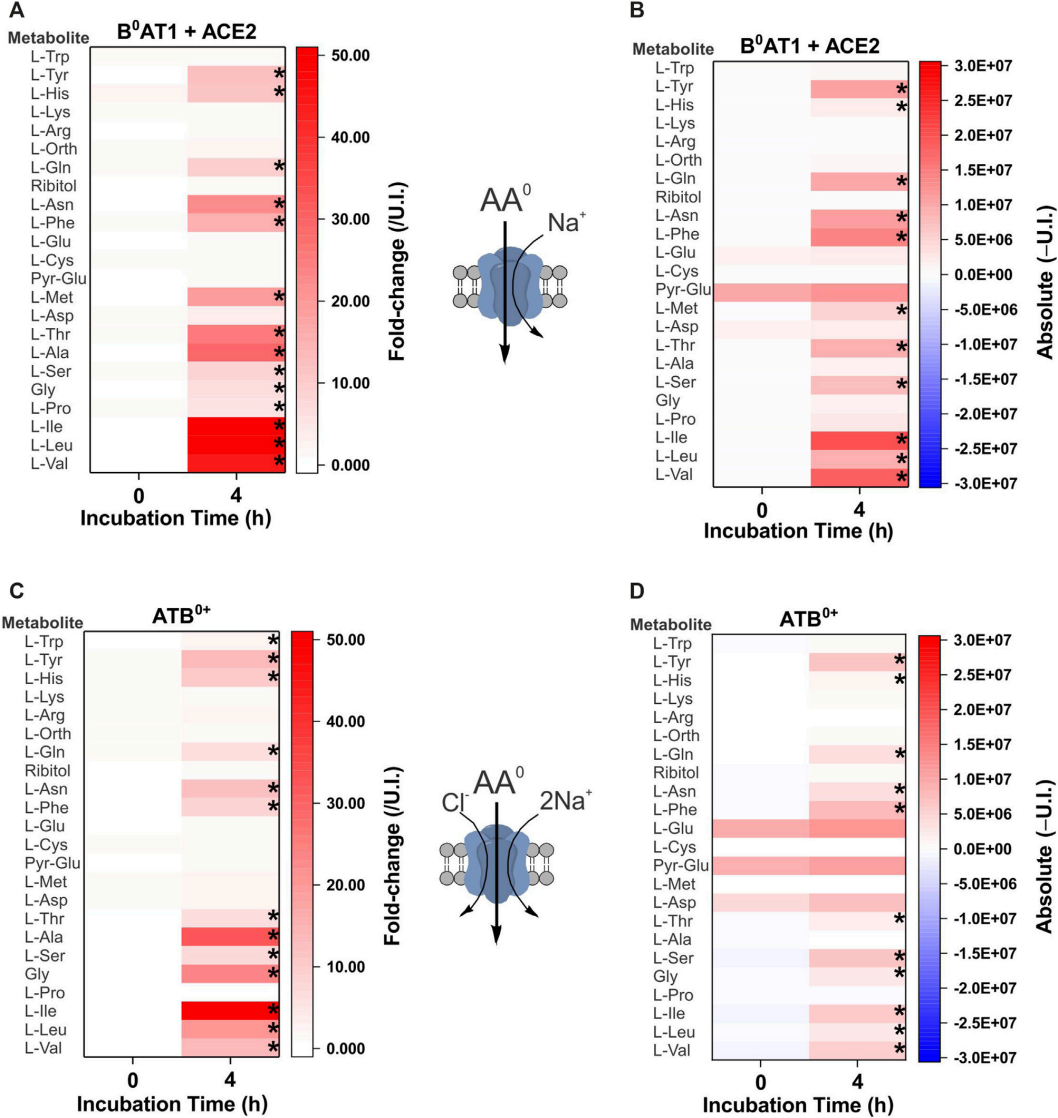
Comprehensive amino acid flux profile of brush border epithelial transporters. Oocytes were each injected with 10 ng cRNA of B^0^AT1 plus ACE2 **(A,B)** or ATB^0,+^ **(C,D)** or remained un-injected (controls). After 4 days of incubation in OR2^+^ (pH 7.8), oocytes were incubated for 4 h in modified L-15 medium before metabolite extraction and analysis. Heat-maps show fold-change **(A,C)** or absolute peak **(B,D)** difference relative to un-injected oocytes. A cartoon of each transporter’s known specificity and stoichiometry is shown between the heat maps. The fold-change **(A,C)** at each time point (0 and 4 h) was calculated by dividing the absolute signal for each amino acid by the corresponding signal from un-injected oocytes, thereby taking account of any natural fluctuations due to endogenous acquisition pathways during incubation. The absolute value heat-maps **(B,D)** were generated by subtracting the signal of un-injected oocytes from the signal in transporter expressing oocytes at each time point (0 or 4 h). A more intense red for the same amino acid at 4 h indicates active accumulation. Pyro-glutamic acid (2-oxo-proline) and ornithine were included in all heat maps as they are known GC-MS derivatives of glutamate and arginine, respectively. The internal standard ribitol was also included in each heat map as a negative control to indicate any deviation from fold-change of 1 ± 0.1. * Indicates a difference in the means between the two time points at the *p* < 0.05 level using a paired *t*-test as calculated from the absolute mass spectrometer raw values. The figures are a representative example of 3 experiments with *n* = 12 transporter-expressing and un-injected oocytes per replicate, *e* = 3.

**FIGURE 4:**
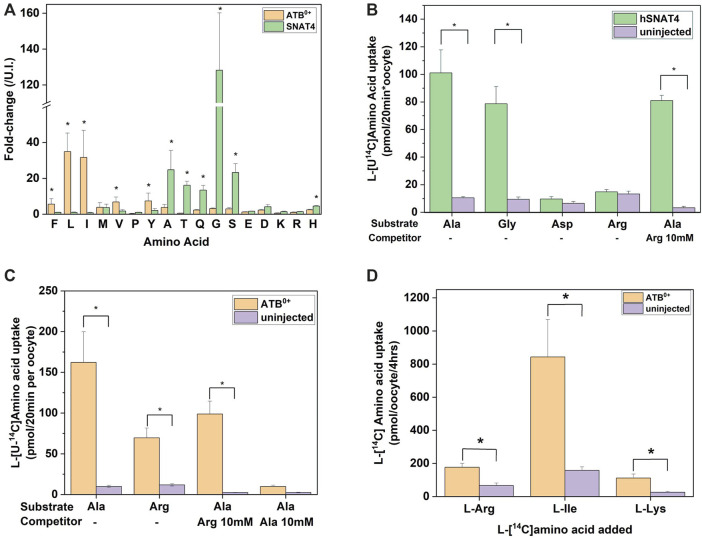
Analysis of SNAT4 and ATB^0+^ transport activity. Oocytes were injected with 10 ng of cRNA for SNAT4 and ATB^0+^ or remained un-injected (controls) for a 4-day expression period in OR2^+^ (pH 7.8). **(A)** Oocytes were incubated for 4 h in modified L-15 medium before sample extraction and analysis by LC-MS relative to un-injected oocytes (U.I.). **(B)** Transport of [^14^C]amino acids was measured over a time period of 20 min in SNAT4 expressing oocytes compared to non-injected oocytes. **(C)** Transport of [^14^C]alanine and arginine was measured over a time period of 20 min in ATB^0+^ expressing oocytes and compared to non-injected oocytes. Where indicated, alanine uptake was challenged by addition of 10 mM unlabelled arginine or alanine. **(D)** Transport of [^14^C]arginine, isoleucine and lysine was measured over a time period of 20 min in ATB^0+^ expressing oocytes and compared to non-injected oocytes. * Indicates a difference in the means between samples at the *p* < 0.05 level using a paired *t*-test as calculated from the absolute mass spectrometer raw values. The figures are a representative example of 3 experiments with *n* = 10 oocytes per repeat.

The second cohort of transporters we analyzed were the apical epithelial symporters B^0^AT1-ACE2 (slc6a19/ACE2) and ATB^0+^ (slc6a14) (Figure 3). The B^0^AT1-ACE2 transporter displayed a substrate profile that included all neutral amino acids (Figure 3A). This was consistent with well-established *in vitro* and *in vivo* profiles (Bohmer et al., 2005; Camargo et al., 2005; Kowalczuk et al., 2008; Singer et al., 2012; Javed and Broer, 2019) with the exception of Trp, a low affinity substrate of B^0^AT1. Due to the low tryptophan concentration in our biomimetic L-15 medium, its transport may have been outcompeted by other amino acids. Analyzing the absolute MS signal also did not reveal a significant increase of tryptophan in B^0^AT1-ACE2 expressing oocytes (Figure 3B). The substrate profile of ATB^0+^ also matched closely to the order of affinity for transporter substrates measured in isolation (Sloan and Mager, 1999), with the significant exception of cationic amino acids arginine, lysine and the neutral amino acid methionine, all of which remained unchanged (Figure 3C). Examining the change in absolute MS signal also did not reveal accumulation of cationic amino acids (Figure 3D). Since arginine and lysine have both previously been reported as substrates of SNAT4 and ATB^0+^ (Sloan and Mager, 1999; Sugawara et al., 2000; Hatanaka et al., 2001), we utilized additional assays to evaluate substrate transport. We also could not rule out that the lack of arginine detection was due to its low R_f_ and incomplete derivatization (Supplementary Table S3), or chemical transformation to ornithine (Leimer et al., 1977; Halket et al., 2005). The lack of cationic AA accumulation by SNAT4-and ATB^0+^-expressing oocytes was nevertheless confirmed by quantitative LC-MS analysis (Figure 4A). LC-MS analysis confirmed glycine, alanine, serine, threonine and glutamine as SNAT4 substrates and revealed a preference of ATB^0,+^ for branched-chain and aromatic amino acids. Using [^14^C]-arginine we were also unable to detect SNAT4-mediated uptake above levels seen with un-injected oocytes (Figure 4B), while [^14^C]-alanine and [^14^C]-glycine were confirmed as substrates of SNAT4. No aspartate uptake was observed, therefore confirming that small increases in GC-MS fold-change analysis most-likely represent *de novo* synthesis. Significant [^14^C]arginine uptake was observed in ATB^0+^-expressing oocytes compared to un-injected controls, however, net uptake was much slower than for the preferred ATB^0+^ substrate alanine (Figure 4C). Notably, when we attempted to out-compete ATB^0+^-mediated [^14^C]alanine uptake with a 100-fold greater concentration of unlabelled arginine, its uptake was only suppressed to the level of uninhibited [^14^C]arginine uptake (Figure 4C). Radiolabeled alanine uptake, by contrast, was totally abolished by a 100-fold excess of unlabelled alanine. We confirmed discrimination against cationic AA transport via ATB^0,+^ by measuring uptake of [^14^C]arginine or [^14^C]lysine in a background of L-15 based incubation medium (Figure 4D). Over 4 h of incubation, accumulation of [^14^C]-cationic amino acids was much lower than that of the high affinity substrate isoleucine.

**FIGURE 5:**
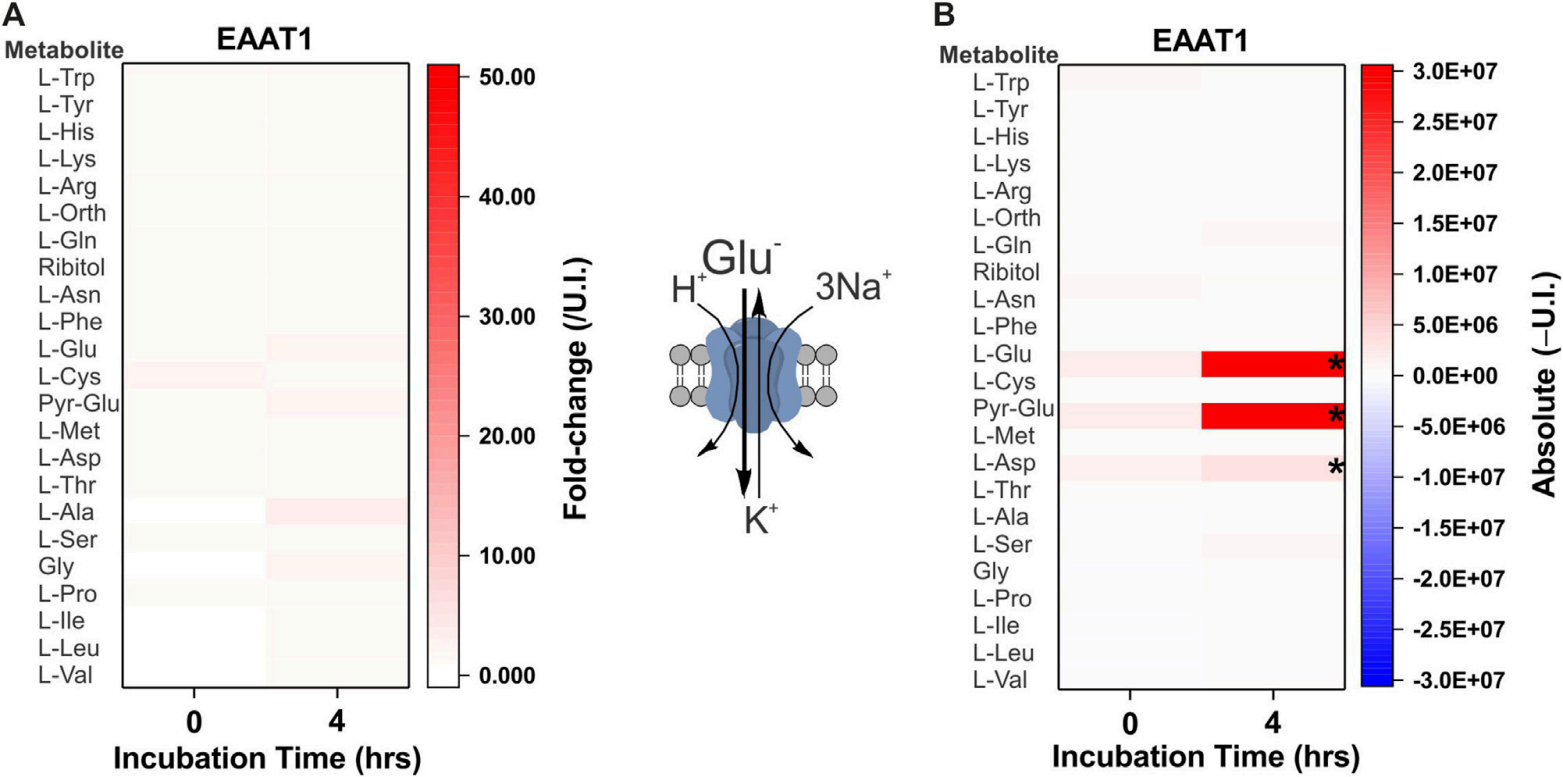
Comprehensive substrate profile of glutamate transporters. Oocytes were injected with 5 ng of cRNA for EAAT1 or remained un-injected for a 4-day expression period in OR2^+^ (pH 7.8). Amino acid accumulation was analyzed as fold-change relative to un-injected (U.I.) oocytes **(A)** or as absolute (Δ) change from U.I. oocytes **(B)** after a 4 h incubation in modified L-15 medium. The known specificity and stoichiometry of the transporters are shown as a cartoon. A more intense red for the same amino acid at 4 h indicates active accumulation. Pyro-glutamic acid (2-oxo-proline) and ornithine are included in all heat maps as they are known GC-MS derivatives of glutamate and arginine, respectively. The internal standard ribitol was also included in each heat-map as a negative control to indicate any deviation from fold-change of 1 ± 0.1 or 10% of the absolute value. * Indicates a difference in the means between the two time points at the *p* < 0.05 level using a paired *t*-test as calculated from the absolute mass spectrometer raw values. The figures are a representative example of 3 experiments with *n* = 12 transporter-expressing and un-injected oocytes per replicate.

As the third cohort we examined glutamate transporters. Due to the high levels of endogenous anionic amino acids, glutamate accumulation was difficult to detect when analyzing fold-change. For instance, the rat brain glutamate/aspartate transporter EAAT1 (slc1a3) showed no significant changes in amino acid accumulation when analyzed by fold-change (Figure 5A), but its activity was revealed when absolute peak areas were compared (Figure 5B).

**FIGURE 6:**
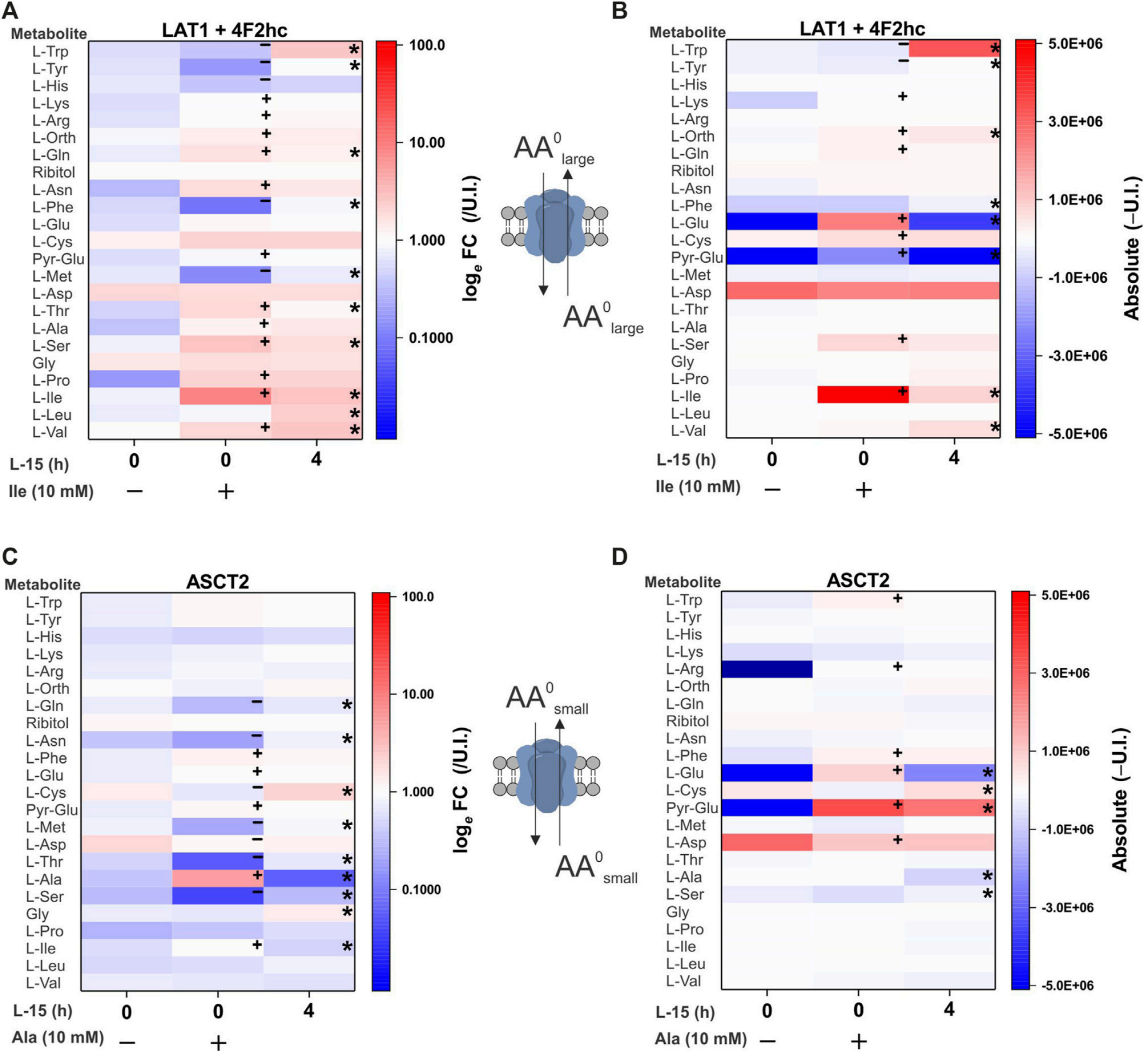
Comprehensive amino acid flux profile of human LAT1-4F2 and ASCT2 exchange transporters. Oocytes were injected with 5 ng each of cRNA for LAT1 and its ancillary protein 4F2hc or 10 ng of ASCT2 cRNA. Injected and un-injected oocytes were maintained for 4 days in OR2^+^ (pH 7.8). In the experiment oocytes were first incubated for 6 h with 10 mM isoleucine **(A,B)** or 10 mM alanine **(C,D)**. Subsequently, oocytes were washed and incubated in modified L-15 medium for 4 h. After each step batches of oocytes were extracted and processed for analysis. Amino acid accumulation was analyzed as fold-change relative to un-injected (U.I.) oocytes **(A,C)** or as absolute (Δ) change from U.I. oocytes **(B,D)**. The known specificity and stoichiometry of the transporters are shown as cartoons. A more intense red for the same amino acid between columns indicates active accumulation, a blue change depletion. Pyro-glutamic acid (2-oxo-proline) and ornithine are included in all heat maps as they are known GC-MS derivatives of glutamate and arginine, respectively. The internal standard ribitol was also included in each heat-map as a negative control to indicate any deviation from fold-change of 1 ± 0.1 or 10% of the absolute value. * Indicates a difference in the means between the two time points at the *p* < 0.05 level using a paired *t*-test as calculated from the absolute mass spectrometer raw values. A + or – sign indicates an increase or decrease of a metabolite during the incubation step. The figures are a representative example of 3 experiments with *n* = 12 transporter-expressing and 12 un-injected oocytes per replicate.

As the final transporter cohort, we analyzed amino acid exchangers. The equilibrium of exchanger-mediated flux is a normalization of all substrate levels on both sides of the membrane without changing the sum of all substrate concentrations. The antiport mechanism rendered our initial attempts to establish a substrate profile unsuccessful, because only very small non-significant variations in intracellular concentrations were observed. To increase the signal, we pre-incubated oocytes with 10 mM of a single substrate in ND96 buffer. During this 6 h preloading period, the chosen amino acid will be imported in exchange for preferred cytosolic amino acids. During the subsequent 4 h medium incubation, the previously preloaded AA will efflux in exchange for preferred extracellular substrates (Figure 6). Each panel has three samples: i) base line before preloading (column 0, −), ii) after 6 h preloading (column 0,+) with 10 mM isoleucine or alanine and iii) after a 4 h incubation in medium (column “4”). Fold-change and absolute-change analysis were conducted after pre-loading and efflux phases to capture low abundance and high abundance amino acid changes. During the preloading phase for the ubiquitous human exchanger LAT1-4F2hc, tryptophan, tyrosine, histidine, phenylalanine and methionine were depleted, while isoleucine increased as expected (Figures 6A,B). The increase of a number of other amino acids during this phase is difficult to explain, particularly of essential amino acids. In the medium incubation phase leucine, valine, tryptophan, phenylalanine and tyrosine increased in abundance, while pre-loaded isoleucine and endogenous glutamine served as major efflux substrates. Histidine appeared to be a good efflux substrate, but its influx was not significant.

An analogous experiment was performed with ASCT2 using alanine as a preloading substrate. During the preloading phase alanine increased, while glutamine, asparagine, cysteine, methionine, threonine and serine were found to be efflux substrates (Figures 6C,D). Due to its low R_f_ in GC-MS, alanine efflux was more easily detected in fold-change analysis than in absolute quantification. Aspartate and glutamate also increased in ASCT2 expressing oocytes during alanine pre-loading, suggesting transamination between alanine, α-ketoglutarate and oxaloacetate. During the medium incubation phase (comparing column 0,+ to column “4”) cysteine, glutamine, asparagine, methionine, threonine, alanine, serine and glycine entered the oocyte as indicated by a change from blue to white or blue to red, while alanine was released (red to blue). These changes could only be detected by fold-change analysis. Absolute quantification, however, suggested that glutamate was an additional efflux substrate. These results demonstrate the ability of our combined metabolomics method to determine the physiological profile and relative substrate specificity of mammalian amino acid transporters with a diverse range of transport mechanisms.

### Identification of SNAT2 and as a Direct Rapid Activator of mTORC1

**FIGURE 7:**
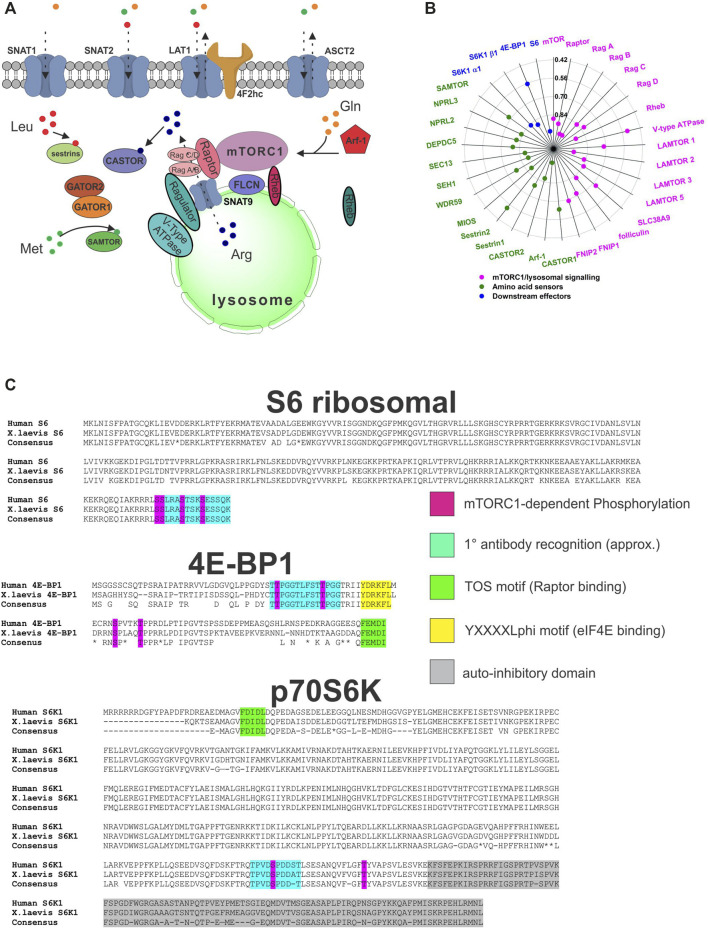
The mTORC1 pathway and amino acid signaling in *Xenopus laevis.* **(A)** Overview of mTORC1 pathway components and associated amino acid sensors and transporters. **(B)** Sequence conservation of *X. laevis* homologues of mTORC1 pathway components. Mean conservation is indicated by a red line. mTORC1 pathway components are divided into 3 groups: the central complex and lysosomal signaling (pink), the upstream amino acid sensing components (green), and the downstream effectors S6K1 (2 isoforms), 4E-BP1, and S6 ribosomal protein (blue). **(C)** Conservation of *X. laevis* mTORC1 effector antibody and phosphorylation sites. Depicted are the peptide sequences of human and *X. laevis* ribosomal protein S6, 4E-BP1, and p70S6K. Areas are indicated for sequences required for anti-human antibody binding–both in non-activated and phosphorylated forms. Conservation is 100% in regions essential for downstream mTORC1 functions as highlighted by the figure legend. The sequence alignment was created by the Clustal Omega algorithm within the HMMER program suite. Sequence accession numbers can be found in Supplementary Table S6.

The role of plasma membrane amino acid transporters in the activation of mTORC1 is unresolved (Condon and Sabatini, 2019; Kim and Guan, 2019). The activation of several downstream mTORC1 signaling components 4E-BP1 and S6K1 has been established in *X. laevis* (Christie et al., 2002)*.* However, many pathway components associated with upstream amino acid sensing and the central mTORC1 complexes remain unreported (Figure 7A). We identified *X. laevis* homologs of all major components of the mTORC1 complex, upstream amino acid sensors and downstream mTORC1 effectors with the exception of the LAMTOR4 protein of the Raptor complex (Supplementary Table S6). In addition, only three Ras-related GTPase (Rag) genes were identified instead of the usual four found in humans and other higher eukaryotes. The average sequence conservation of *X. laevis* mTORC1 amino acid sensing components to those in human homologues was 81.2% (Figure 7B), which was much higher than the global sequence conservation average for all ORFs (Session et al., 2016). Phosphorylation sites in the downstream mTORC1 effectors p70SK1, S6 and 4E-BP1, were conserved as was, crucially for protein detection, the epitope recognition region of anti-human antibodies (Figure 7C). The conservation of antibody recognition epitopes in *X. laevis* mTORC1 components allows for their detection by western blotting. Using these combined results we hypothesized that mTORC1 is functional in *X. laevis* cells and that phosphorylation of downstream substrates ribosomal S6 and 4E-BP1 will occur in a similar manner as in mammalian cells. This is in agreement with sequence comparisons made by other groups (Wolfson and Sabatini, 2017) (Tatebe and Shiozaki, 2017) and the response of mTORC1 in Drosophila S2 cells to amino acids (Zoncu et al., 2011).

**FIGURE 8:**
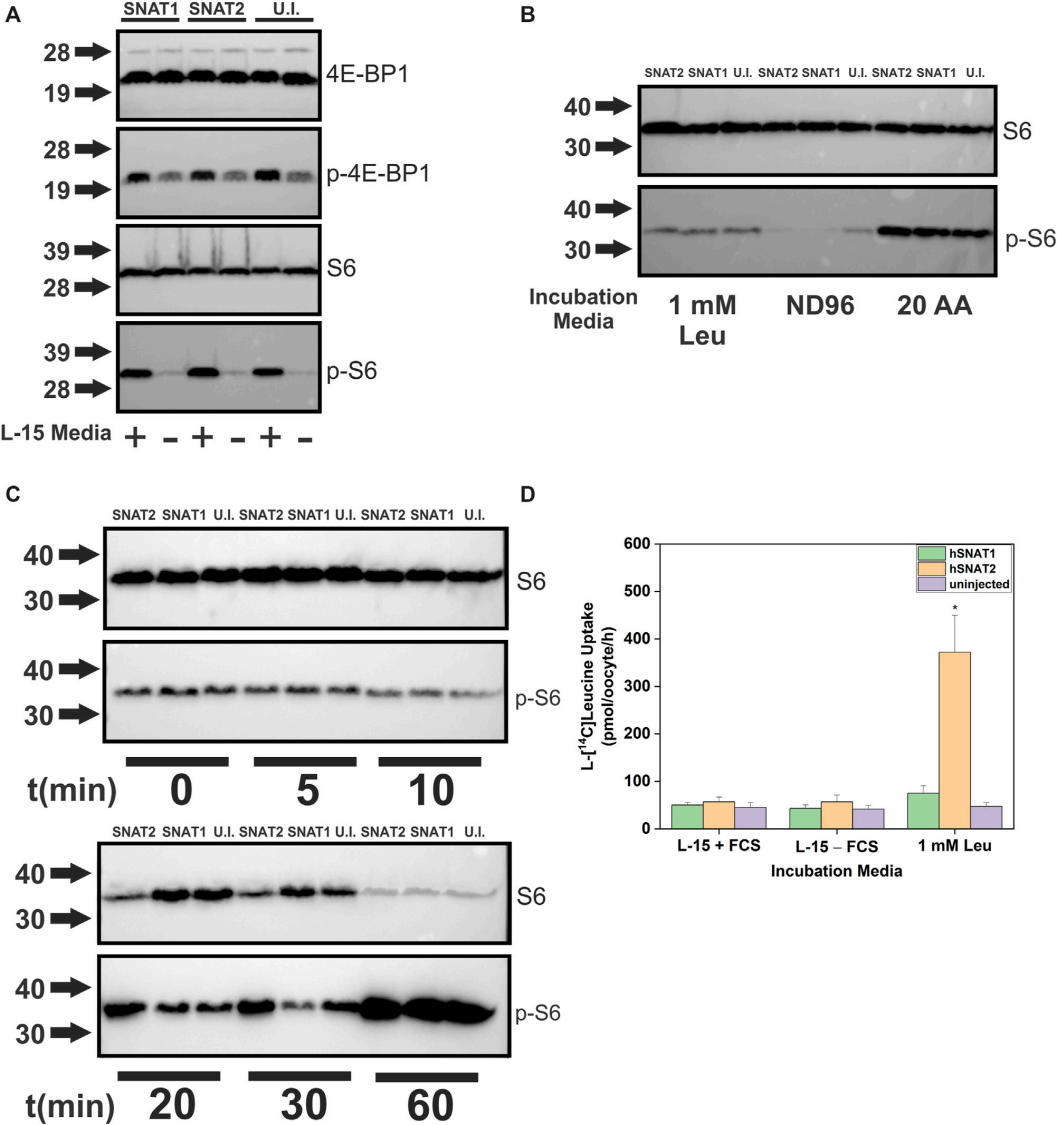
Human SNAT2 but not SNAT1 is an activator of mTORC1. Oocytes were injected with 10 ng cRNA of either SNAT1or SNAT2. After 4 days incubation in OR2^+^ (pH 7.8), oocytes were incubated in modified L-15, 1  ×  ND96 (pH 7.4) or a mix of all 20 AA in ND96. The activation of mTORC1 targets was ascertained by immunodetection of S6/phospho-S6 and 4E-BP1/phospho-4E-BP1. **(A)** Changes of 4E-BP1 and S6 phosphorylation in *X. laevis* oocytes incubated in L-15 biomimetic media or 1  ×  ND96 (pH 7.4) over 4 h. **(B)** Changes to the phosphorylation state of ribosomal protein S6 by a mixture of 20 proteinaceous amino acids, or leucine alone after 4 h incubation. Amino acids were added at a concentration of 1 mM each. **(C)** Time course of S6 phosphorylation after incubation in 20 × AA (1 mM each) mixture in oocytes expressing SNAT1, SNAT1 or un-injected oocytes. **(D)** Uptake of 1 mM L-[^14^C]leucine was measured in the presence of L-15 biomimetic media, 20 × AA or 1 mM leucine alone over 4 h. * Indicates a difference in the means between oocytes expressing SNAT2 and those expressing SNAT1 or un-injected oocytes in the same incubation media (*p* < 0.05, *n* = 10 oocytes, 3 experimental repeats).

**FIGURE 9:**
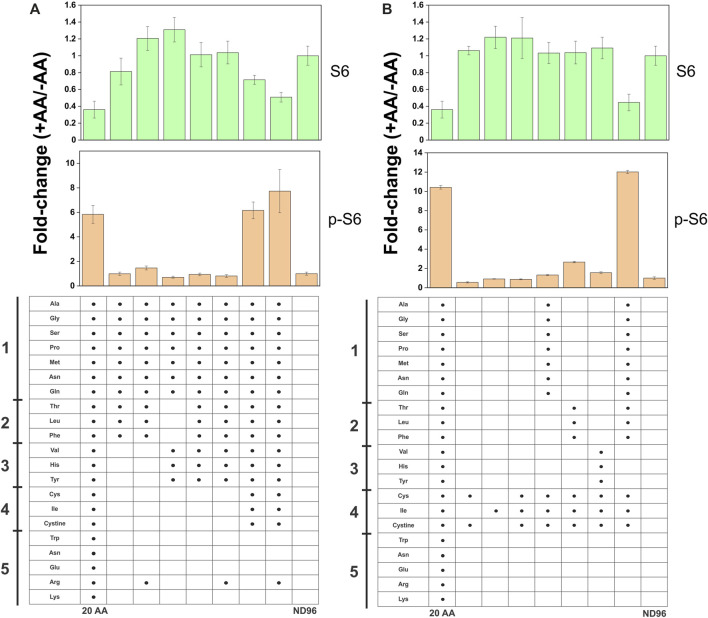
Complex amino acid requirements for full activation of mTORC1 in oocytes. Un-injected oocytes were incubated for 4 days in OR^2+^ (pH 7.8) buffer. In the experiment, oocytes were incubated with different amino acid mixtures (1 mM each) for 4 h. Activation of mTORC1 was measured by immunodetection of ribosomal protein S6 and phospho-S6. Densitometry in each bar graph was normalized to the negative control, where oocytes were incubated without amino acids in 1 × ND96 buffer only (last column). Bar graphs represent the mean ± S.E.M. of 3 experimental repeats (*e* = 3) with each condition in each repeat representing the protein expression of 12 oocytes (*n* = 12). The amino acid composition of each group shown below the densitometry bar graphs was based on several criteria: substrate affinity for SNAT2, previously shown ability to activate mTORC1, and amino acids previously shown to activate mTORC1 when injected into oocytes (Christie et al., 2002). Amino acid groups are indicated by numbers 1, 2, 3, 4, 5 in the margin. The graph was divided into panels **A and B** for clarity.

The transporter substrate profiles and conservation of amino acid sensing components in *X. laevis* allowed us to analyze transporters as potential mTORC1 activators (Figure 8A). SNAT2 was particularly interesting as our profile demonstrated its ability to accumulate a significant amount of cytosolic leucine, a known activator of mTORC1, in contrast to its close paralog SNAT1 (Figures 2A,B). Anti-human antibodies for 4E-BP1, phospho-4E-BP1, ribosomal protein S6 and phospho-S6 were able to detect *Xenopus* orthologues and their phosphorylation states upon incubation in modified L-15 (Figure 8A). Using a 4 h incubation period, heterologous expression of SNAT1 and SNAT2 did not affect mTORC1 signaling. In all subsequent immunoblot experiments we used phospho-S6 as the most reliable read-out of mTORC1 activation. To identify the minimal nutrient requirements needed for mTORC1 activation we replaced L-15 biomimetic medium with ND96 buffer, which abolished mTORC1 activation (Figure 8B). Re-introducing leucine alone only partially activated mTORC1 after 4 h incubation, confirming previous reports that leucine or leucine plus arginine alone cannot fully activate the pathway in the absence of other amino acids (Hara et al., 1998). This reflects the endogenous expression of oocyte AA transporters and the subsequent ability of oocytes to acquire amino acids sufficient to activate endogenous mTORC1 signaling. Accordingly, we reasoned that transporter-expressing oocytes with the capacity to achieve activation of mTORC1 would do so more rapidly than un-injected oocytes, whether through accumulation of amino acids or transceptor activity. Over a 60 min time-course (Figure 8C), SNAT2 demonstrated an advanced capacity to activate mTORC1, as evidenced by a significant increase in phospho-S6 between 10 and 30 min with a concurrent decrease in S6. By contrast, SNAT1-expressing oocytes activated mTORC1 only as well as un-injected oocytes. This result was notable as SNAT1 displays little difference in its substrate profile from SNAT2 apart from the latter’s significantly greater capacity to accumulate leucine and proline (compare Figures 2A,B). Given that leucine alone cannot fully activate mTORC1 in oocytes (Figure 8B) the result suggests that a combination of small neutral amino acids plus leucine may rapidly activate mTORC1 in oocytes. Activation of mTORC1 occurred at time-points where accumulation of leucine was marginal (Figure 8D). Given that heterologously expressed transporters were not essential to activate mTORC1 in oocytes, we divided amino acids into five groups to determine the optimal mixture required for mTORC1 activation (Figure 9). It was apparent that numerous amino acids were necessary for full mTORC1 activation. The results confirmed that a mix of mainly SNAT2 substrates (alanine, serine, glycine, proline, methionine, asparagine, glutamine, threonine, leucine, phenylalanine, valine, histidine, tyrosine, cysteine, isoleucine, cystine) optimally activated mTORC1 (Figures 9A,B). Notably extracellular arginine was not required for full activation in *X. laevis* oocytes.

## Discussion

We have successfully developed a combined single-cell GC-MS/LC-MS method for elucidating the substrate profiles of multiple membrane transporters. The potential of this technique is shown by the substrate profiles we have established for several amino acid transporters in a physiologically relevant environment. The method readily demonstrates the capability of Na^+^-dependent symporters to load amino acids into a cell. The results largely confirm findings from numerous previous studies in which a mix of active transport and competition was used to determine substrate preference of amino acid transporters. More prominently than previously shown (Yao et al., 2000), we were able to detect that SNAT2 is very capable of accumulating leucine and proline compared to SNAT1 (Yao et al., 2000; Albers et al., 2001; Chaudhry et al., 2002; Mackenzie and Erickson, 2004). We could also confirm that SNAT4 has a narrower substrate specificity than SNAT1 and SNAT2, excluding larger polar neutral amino acids. However, we could not detect the accumulation of cationic amino acids by SNAT4, as reported earlier (Hatanaka et al., 2000). While we could detect the uptake of labeled cationic amino acids originally reported for human ATB^0+^ (Sloan and Mager, 1999), these were not accumulated in oocytes incubated in a complex matrix with the mouse homologue used in this study. Arginine-dependent effects in colon organoids were abolished in ATB^0,+^ ko organoids (Ahmadi et al., 2018), but the amino acid content of the colon lumen is unknown. Accumulation of neutral amino acids is most readily detected as evidenced by the substrate specificity of B^0^AT1. The only exception for this transporter was tryptophan, the transport of which has been verified *in vivo* (Singer et al., 2012; Javed and Broer, 2019), but was hardly detectable in oocytes. Tryptophan is present in low concentration in L-15 medium, and thus it may have been out-competed by other amino acids. For ATB^0+^ this explains the lack of observed methionine accumulation at its relatively low concentration in L-15. Antiporters or exchangers can also be profiled using this method. However, we had to modify the method because the sum of their substrate concentrations remains the same on both sides of the membrane and neutral amino acid concentrations are fairly balanced. To detect antiport activity, an imbalance was introduced by incubating the oocytes with one dominant amino acid substrate in order to trans-stimulate movement of intracellular antiporter substrates. Using isoleucine we could detect tryptophan, tyrosine, histidine, phenylalanine, methionine, leucine, valine as LAT1 substrates, which is in agreement with previous studies (Yanagida et al., 2001; Morimoto et al., 2008). Glutamine was also detected as an efflux substrate. For ASCT2, we used alanine as the pre-loading substrate. Glutamine, asparagine, cysteine, methionine, threonine and serine were found to be efflux substrates at neutral pH, while alanine was accumulated. During medium incubation cysteine, glutamine, asparagine, methionine, threonine, serine and glycine entered the oocyte, while alanine was the main efflux substrate. The transport of cysteine refutes some of the data using reconstituted ASCT2 in proteoliposomes, which suggested that cysteine is an allosteric modulator of the transporter (Scalise et al., 2015). We could confirm low-affinity transport of glutamate by ASCT2 (Scalise et al., 2020), which is only visible in the efflux direction, because intracellular glutamate is ∼2 mM. A common feature of incubation in L-15 media over extended time periods is the increase in intracellular anionic amino acids, particularly aspartate. These increases occur in oocytes that are not expressing transporters but also occur more rapidly in those that do. The most likely explanation is the transamination of preloaded amino acids generating aspartate and glutamate. When using GC-MS to accurately identify the full biologically relevant substrate specificity, both fold-change and absolute values should be used during the analysis of initial experiments. Traditional GC-MS and LC-MS based metabolomics analysis rely on relative fold-changes to identify underlying important metabolic changes–an approach that can lead to potential false negatives if not accompanied by additional absolute signal analysis. Our quantification of endogenous amino acid concentrations in oocytes using GC-MS largely agreed with the value range previously established using HPLC in stage 5 or 6 oocytes (Taylor and Smith, 1987; Meier et al., 2002). The exception of glutamate can be explained by the inability to distinguish glutamate and glutamine in these previous studies, which, adding the two together, may over-estimate the glutamate concentration values provided. The moderately higher concentrations of isoleucine, proline, methionine and tyrosine we measured are more difficult to explain. However, large variations of amino acid concentrations have been noted (Taylor and Smith, 1987; Meier et al., 2002) between oocyte stages, time post-surgery, and ionic incubation conditions.

The method developed here can also be used to investigate amino acid signaling, the components of which are highly conserved in multicellular eukaryotes (Panchaud et al., 2013; Saxton and Sabatini, 2017; Tatebe and Shiozaki, 2017; Javed and Fairweather, 2019). Our results support the concept of SNAT2 as a transceptor (Pinilla et al., 2011), because mTORC1 activation was observed at time points where cytosolic leucine was barely increased. Another SNAT2 substrate glutamine has also been shown to activate mTORC1 in HepG2 and HeLa cells independent of the cytosolic Leu concentration, also suggesting SNAT2 can activate mTORC1 as a transceptor using glutamine (Chiu et al., 2012). Using groups of amino acids to activate mTORC1 in un-injected oocytes, we found an intriguing coincidence between the substrates of SNAT2 and their ability to activate mTORC1. The extended N-terminus of slc38 member SNAT9 has recently been shown to bind to the RagA/C heterodimer (Rebsamen et al., 2015; Wang et al., 2015; Fromm et al., 2020) and occupy the arginine binding site during mTORC1 inactivation (Lei et al., 2018; Lei et al., 2020). A similar mechanism could be envisioned for the N-terminus of SNAT2 (Gaccioli et al., 2006; Hundal and Taylor, 2009; Poncet and Taylor, 2013), although the N-terminal is predicted to be 50 residues shorter than that of SNAT9. We cannot discount, however, the activation of mTORC1 through the accumulation of cytosolic amino acids, particularly but not exclusively leucine, mediated by SNAT2 (Wolfson et al., 2016). Alternatively, SNAT2 could mediate accumulation of amino acids via tertiary active transport involving endogenously expressed transporters which then could activate mTORC1. Our results support the notion that SNAT2 represents a likely candidate as an important mTORC1 activator/accelerator as has been previously proposed (Hyde et al., 2007; Baird et al., 2009; Pinilla et al., 2011; Poncet and Taylor, 2013; Broer and Broer, 2017; Broer, 2018; Hoffmann et al., 2018). The activation of mTORC1 by SNAT2 represents an alternative mechanism to the long-suggested combined action of the amino acid transporters LAT1 and ASCT2 (Nicklin et al., 2009). ASCT2, in particular, has recently been shown to be dispensable for mTORC1 activation due to compensation by SNAT2 (Broer et al., 2016). Key to the ability of SNAT2 to activate mTORC1 in our experiments is the capacity for leucine accumulation, which has been suggested but never directly shown to be a significant SNAT2 substrate previously (Hatanaka et al., 2000; Gazzola et al., 2001; Tang et al., 2018). The method outlined here can be applied to test the ability of other mammalian amino acid transporters, or combination of transporters, to activate mTORC1, including the frequently cited combination of LAT1 and ASCT2. Our method can also be applied to mammalian cells but would require genetic or pharmacological tools to inhibit endogenous transporters. Mammalian cell lines may express between 8 and 25 amino acid transporters at the plasma membrane–a significant proportion of which would have to be disrupted in order to isolate a specific transporter (Gauthier-Coles et al., 2021). In oocytes, proteomic and transcriptomic data demonstrate the presence of amino acid transporter paralogues of SLC1A5 (ASCT2), SLC3A2, SLC6A7 (PROT), SLC6A14 (B^0,+^AT), SLC7A9 (b^0,+^AT), SLC7A4 (CAT4, lysosomal), SLC36A1 (PAT1, lysosomal), SLC36A4 (PAT4), SLC38A9 (lysosomal) and SLC38A10 (ER) (Smits et al., 2014; Wühr et al., 2014). Despite many-fold higher expression of heterologous mRNA, silencing of endogenous oocyte transporters may increase the signal by slowing the endogenous activation of mTORC1. In this study we have not systematically investigated the minimal concentration of amino acids required to activate mTORC1 in *X. laevis* oocytes. Further investigation will be required to determine these minimal activating concentrations.

This study also represents the first comprehensive metabolic profiling of *X. laevis* oocytes, a widely used experimental system for membrane protein physiology and as a model in developmental biology. Moreover, we demonstrated, as suspected previously (Taylor and Smith, 1987; Broer, 2010; Sobczak et al., 2010) that oocytes are not metabolically silent. All oocyte incubations for 4 h in our L-15 media matrix led to increases in TCA cycle intermediates and anionic amino acids independent of the transporter expressed, suggesting the use of neutral amino acids as anaplerotic substrates. We also suggest that decreases in multiple intracellular amino acids observed following transporter cRNA injection into oocytes (e.g., Figure 6) probably represent their use for protein synthesis.

In summary we show that single-oocyte metabolomics is a useful tool to explore the nexus between amino acid transport, amino acid homeostasis and signaling. The method can be applied to other types of transporters and may also be useful to explore the activity of organellar transporters and additional transporter-regulated intracellular signaling pathways.

## Data Availability

The data presented in the study are deposited in the MetaboLights repository (https://www.ebi.ac.uk/metabolights), accession number MTBLS2476.
