# Supplementary material for: A GC-MS/Single-Cell Method to Evaluate Membrane Transporter Substrate Specificity and Signaling
Source: Front Mol Biosci. 2021 Apr 13;8:646574. doi: 10.3389/fmolb.2021.646574 (PMC8076599; doi:10.3389/fmolb.2021.646574)
Supplement: Supplementary file 1 [file datasheet1.docx]

# Supplementary Tables

**Table S1: Membrane amino acid transporters used in this study**

| **Protein** | **Gene Name** | **Species** | **Function** | **Cloning References** |
| --- | --- | --- | --- | --- |
| SNAT1 | SLC38A1 | Human | Sodium-dependent neutral amino acid, sodium-independent cationic amino acid acquisition | (Broer et al., 2016) |
| SNAT2 | SLC38A2 | Human |  | (Broer et al., 2016) |
| SNAT4 | SLC38A4 | Human |  | (Broer et al., 2016) |
| B^0^AT1 + ACE2 | SLC6A19 | Human | Sodium-dependent small intestinal and kidney neutral amino acquisition | (Seow et al., 2004) |
| ATB^0+^ | SLC6A14 | Mouse | Sodium- and chloride-dependent large intestine neutral/cationic amino acquisition | This study |
| EAAT1 (GLAST) | SLC1A3 | Rat | Neuronal glial cell reuptake of glutamate and L/D-aspartate | (Bröer et al., 1999) |
| ASCT1 | SLC1A4 | human | Small neutral L-amino acid exchanger & D-serine | This study |
| ASCT2 | SLC1A5 | Human | Neutral amino acid exchanger | (Broer et al., 2016) |
| LAT1-4F2hc | SLC7A5 + SLC3A2 | Rat + Human | Large neutral amino acid exchanger and multiple other metabolites | (Wagner et al., 2000) |
| y^+^LAT2-4F2hc | SLC7A6 + SLC3A2 | Human | Arg^+^ or Leu/Gln + sodium exchanger | (Chubb et al., 2006) |

**Table S2**: **Composition of L-15-based and other in-house incubation matrices**

| **Major Component** | **Individual components** | **[component]_final_ (mM)** |
| --- | --- | --- |
| **L-15 Matrix** | | |
| L-15 Amino Acids^†^ | L-Asn (anhydrous) | 1.21$-$1.88 |
|  | L-Ser | 1.22$-$1.90 |
|  | L-Thr | 1.61$-$-2.51 |
|  | L-Tyr (free base) | 1.06$-$1.65 |
|  | L-Ala | 1.62$-$2.52 |
|  | L-Val | 0.546$-$0.850 |
|  | L-Met | 0.322$-$0.501 |
|  | L-Ile | 0.610$-$0.950 |
|  | L-Leu | 0.610$-$0.950 |
|  | L-Phe | 0.484$-$0.753 |
|  | L-His | 1.03$-$1.61 |
|  | L-Lys monohydrochloride | 0.328$-$0.510 |
|  | L-Arg (free base) | 1.84$-$2.86 |
|  | L-Cys (free base) | 0.664$-$1.03 |
|  | Gly | 1.71$-$2.66 |
|  | L-Trp | 0.063$-$0.098 |
| L-15 salts | CaCl_2_•2H_2_O | 0.808$-$1.258 |
|  | MgCl_2_•6H_2_O | 0.631$-$0.984 |
|  | MgSO_4_ (anhydrous) | 0.521$-$0.811 |
|  | KCl | 3.45$-$5.37 |
|  | KH_2_PO_4_ (anhydrous) | 0.283$-$0.441 |
|  | NaCl | 87.9$-$137 |
|  | Na_2_HPO_4_ (anhydrous) | 0.859$-$1.338 |
| L-15 Vitamin | Choline Chloride | 4 $\times$ 10^-3^$-$7 $\times$ 10^-3^ |
|  | Flavin Mononucleotide•Na | 1.4 $\times$ 10^-4^$-$2.1 $\times$ 10^-4^ |
|  | Folic Acid | 1.5 $\times$ 10^-3^$-$2.2 $\times$ 10^-3^ |
|  | myo-Inositol | 7 $\times$ 10^-3^$-$0.011 |
|  | Niacinamide | 3 $\times$ 10^-3^$-$5 $\times$ 10^-3^ |
|  | DL-Pantothenic Acid•½Ca | 2.9 $\times$ 10^-3^-4.6 $\times$ 10^-3^ |
|  | Pyridoxine•HCl | 4.9 $\times$ 10^-3^$-$3.1 $\times$ 10^-3^ |
|  | Thiamine Monophosphate•HCl | 1.7 $\times$ 10^-3^$-$2.6 $\times$ 10^-3^ |
| L-15 other components | D-Galactose | 3.21$-$5.00 |
|  | Galactose Oxidase | 7.6 $\times$ 10^-3^$-$0.012 |
|  | Phenol Red•Na | 0.019$-$0.029 |
|  | Pyruvic Acid•Na | 3.21$-$5.00 |
| FCS (Gibco) | Many | $-$ |
| 4 $\times$ amino acid mix | L-Gln | 2 |
|  | L-Asp | 6 $\times$ 10^-3^ |
|  | L-Glu | 0.360 |
|  | L-Pro | 0.098 |
| HEPES | HEPES free-acid | 20 |
| MilliQ H_2_O dilution | 7:10:33 (H_2_O:FCS:L-15) | |

†All concentrations from L-15 Media components are given as a range. With the lower limit calculated as the concentration if L-15 diluted with other ingredients containing no amino acids. The upper limit is taken as if no dilution had occurred (i.e. the concentration from the starting L-15 media). This is due to the concentration of components in FCS being unknown but assumed to be equivalent to blood plasma concentrations, the actual final concentrations of all these components will lie closer to the higher limit.

**Table S3: TMS-derivatisation of canonical amino acids and selected metabolites isolated from *X.laevis* oocytes**

| **Metabolite** | **1 TMS Derivative (%)^†^** | **2 TMS Derivative (%)^†^** | **3 TMS Derivative (%)^†^** | **4 TMS Derivative (%)^†^** | $\sum\boldsymbol{d}_{\boldsymbol{EIC}}$^Ⱶ^ |
| --- | --- | --- | --- | --- | --- |
| Valine | $-$ | 100 | $-$ | $-$ | 3.93 $\times$ 10^6^ |
| Leucine | $-$ | 100 | $-$ | $-$ | 3.23 $\times$ 10^5^ |
| Isoleucine | 8.1 | 91.9 | $-$ | $-$ | 2.29 $\times$ 10^6^ |
| Proline | 19.1 | 80.9 | $-$ | $-$ | 8.72 $\times$ 10^4^ |
| Glycine | $-$ | $-$ | 100 | $-$ | 1.66 $\times$ 10^6^ |
| Serine | $-$ | 29.5 | 70.5 | $-$ | 1.77 $\times$ 10^7^ |
| Alanine | $-$ | $-$ | 100 | $-$ | 3.35 $\times$ 10^4^ |
| Threonine | $-$ | 26.8 | 73.2 | $-$ | 8.29 $\times$ 10^6^ |
| Aspartic acid | $-$ | 25.5 | 74.5 | $-$ | 7.81 $\times$ 10^7^ |
| Methionine | 16.2 | 83.8 | $-$ | $-$ | 2.29 $\times$ 10^6^ |
| Pyro-glutamate (2-oxo-Proline) |  | 100 | $-$ | $-$ | 3.28 $\times$ 10^6^ |
| Cysteine | $-$ | $-$ | 100 | $-$ | 3.93 $\times$ 10^5^ |
| Glutamic acid | $-$ | 0.27 | 99.73 | $-$ | 8.01 $\times$ 10^7^ |
| Phenylalanine | 6.4 | 93.6 | $-$ | $-$ | 2.21 $\times$ 10^5^ |
| Asparagine | $-$ | 8.4^*^ | 91.6 | $-$ | 5.45 $\times$ 10^5^ |
| Glutamine | $-$ | 1.2 | 94.0^‡^ | 4.8 | 5.78 $\times$ 10^6^ |
| Ornithine | $-$ | $-$ | 18.2^♯^ | 81.8 | 9.83 $\times$ 10^6^ |
| Arginine | $-$ | $-$ | 100 | $-$ | 9.87 $\times$ 10^5^ |
| Lysine | $-$ | $-$ | 1.4^♯^ | 98.6 | 1.67 $\times$ 10^7^ |
| Histidine | $-$ | $-$ | 100 | $-$ | 2.36 $\times$ 10^6^ |
| Tyrosine | $-$ | 2.0 | 98.0 | $-$ | 1.148 $\times$ 10^7^ |
| Tryptophan | $-$ | $-$ | 100 | $-$ | 2.95 $\times$ 10^6^ |

† Relative to the total TMS derivative species base peak EIC integration

**Ⱶ** $\sum\boldsymbol{d}_{\boldsymbol{EIC}}$ total EIC base peak integration of TMS derivatives; calculated from uninjected oocyte batch (see Table S4).

‡ Does not include glutamine $-$H_2_O (3TMS) species (see Table S4), which is detected but gives a negligible signal.

* Includes both asparagine $-$H_2_O (2TMS) and asparagine (2TMS) species (see Table S4).

♯ Includes 2 $\times$ 3TMS derivatives identified by the GOLM database (see Table S4).

**Table S4: Metabolites isolated from *X.laevis* oocytes by GC-MS aqueous phase extraction**

| **Metabolite** | **R.I. (Kovats)^Ⱶ^** | **Average R.T. (min)^Ⱶ^** | **Quantification ion (m/z)** | **QC Relative S.D.^†^ (%)** | **TMS derivative** | **ID Criteria^‡^** |
| --- | --- | --- | --- | --- | --- | --- |
| Isoleucine | 1180.0 | 6.494 | 86 | 23.2 | 1 | 2 |
| Proline | 1176.0 | 6.491 | 70 | 14.3 | 1 | 2 |
| Phosphate | 1171.6 | 6.420 | 241 | 25.6 | 2 | 1 |
| Glycine | 1194.5 | 6.620 | 228 | 31.2 | 1 | 3 |
| Valine | 1208.1 | 6.741 | 144 | 12.8 | 2 | 1 |
| Urea | 1241.4 | 7.033 | 189 | 8.7 | 2 | 1 |
| Serine | 1252.9 | 7.133 | 116 | 42.3 | 2 | 1 |
| Glycerol | 1259.3 | 7.189 | 205 | 22.7 | 3 | 1 |
| Ethanolamine | 1260.1 | 7.196 | 174 | 18.0 | 3 | 1 |
| Leucine | 1264.8 | 7.237 | 158 | 15.4 | 2 | 2 |
| Phosphoric acid | 1263.4 | 7.225 | 299 | 11.5 | 3 | 1 |
| 2-deoxy-Ribofuranose | 1270.7 | 7.289 | 170 | 25.4 | 2 | 1 |
| Diethanolamine | 1277.9 | 7.353 | 146 | 34.9 | 2 | 1 |
| Isoleucine | 1286.4 | 7.427 | 158 | 21.3 | 2 | 2 |
| Threonine | 1290.3 | 7.461 | 117 | 26.8 | 2 | 2 |
| Proline | 1297.2 | 7.521 | 142 | 10.7 | 2 | 2 |
| Glycine | 1302.6 | 7.569 | 174 | 10.2 | 3 | 2 |
| Nicotinic acid | 1303.9 | 7.580 | 180 | 7.5 | 1 | 1 |
| Succinic acid (butanedioic acid) | 1309.2 | 7.626 | 247 | 12.8 | 2 | 1 |
| Glyceric acid (propanoic acid) | 1318.4 | 7.708 | 189 | 19.8 | 3 | 1 |
| Uracil | 1335.6 | 7.858 | 241 | 15.5 | 2 | 1 |
| Fumaric acid (2-butenedioic acid) | 1345.0 | 7.940 | 245 | 10.1 | 2 | 1 |
| Serine | 1349.4 | 7.979 | 204 | 28.1 | 3 | 1 |
| Alanine | 1359.5 | 8.068 | 188 | 39. 4 | 3 | 2 |
| 3-cyano-alanine | 1368.9 | 8.150 | 141 | 22.0 | 2 | 1 |
| Threonine | 1374.8 | 8.202 | 218 | 24.4 | 3 | 2 |
| Cadaverine | 1391.6 | 8.349 | 174 | 29.8 | 4 | 3 |
| Thymine | 1397.2 | 8.399 | 255 | 3.4 | 2 | 1 |
| Hydroquinone | 1401.1 | 8.433 | 239 | 17.8 | 2 | 3 |
| Methionine | 1418.4 | 8.584 | 104 | 20.2 | 1 | 2 |
| Aspartic acid | 1421.1 | 8.608 | 160 | 15.9 | 2 | 2 |
| β-alanine | 1423.1 | 8.625 | 174 | 21.8 | 3 | 1 |
| 5-hydroxy-lysine | 1445.1 | 8.818 | 174 | 12.4 | 4 | 1 |
| Ornithine-1,5,-lactam | 1455.0 | 8.905 | 128 | 20.7 | 2 | 1 |
| Glutamine (-H_2_O) | 1469.1 | 9.029 | 155 | 25.8 | 2 | 2 |
| Malic acid | 1474.1 | 9.073 | 233 | 9.8 | 3 | 1 |
| Threitol | 1487.2 | 9.188 | 217 | 17.0 | 4 | 1 |
| Nicotinamide | 1491.2 | 9.223 | 179 | 13.9 | 1 | 1 |
| Asparagine (-H_2_O) | 1501.4 | 9.310 | 243 | 19.6 | 2 | 2 |
| Aspartic acid | 1508.0 | 9.360 | 232 | 16.8 | 3 | 2 |
| Adipo-2,6-lactam | 1513.6 | 9.402 | 170 | 32.4 | 2 | 1 |
| Methionine | 1515.7 | 9.418 | 176 | 27.0 | 2 | 2 |
| Pyroglutamic acid/5-oxo Proline | 1523.9 | 9.482 | 156 | 8.2 | 2 | 2 |
| Glutamic acid | 1527.7 | 9.508 | 174 | 10.8 | 2 | 2 |
| Cysteine | 1550.0 | 9.675 | 220 | 38.2 | 3 | 2 |
| Creatinine | 1555.3 | 9.712 | 115 | 15.2 | 3 | 1 |
| Phenylalanine | 1560.7 | 9.755 | 120 | 23.4 | 1 | 2 |
| α-Ketoglutaric acid (2-Oxoglutaric acid) | 1568.6 | 9.786 | 198 | 28.9 | 2 (1 MeOx) | 1 |
| 3-phenyl-lactic acid (phenyllactate) | 1587.3 | 9.955 | 193 | 14.0 | 2 | 1 |
| Glutamine (-H_2_O) | 1589.9 | 9.974 | 227 | 40.7 | 3 | 2 |
| Asparagine | 1602.2 | 10.067 | 159 | 8.2 | 2 | 2 |
| Ornithine | 1609.8 | 10.123 | 142 | 29.2 | 3 | 2 |
| Glutamic acid | 1616.1 | 10.171 | 246 | 12.7 | 3 | 2 |
| Phenylalanine | 1633.9 | 10.304 | 218 | 11.3 | 2 | 2 |
| Pyrophosphate | 1653.8 | 10.454 | 451 | 29.5 | 4 | 1 |
| Homocysteine | 1663.8 | 10.528 | 234 | 32.2 | 3 | 1 |
| Asparagine | 1669.1 | 10.568 | 231 | 14.3 | 3 | 2 |
| Taurine (2-aminoethanesulfonic acid) | 1674.7 | 10.611 | 326 | 14.7 | 3 | 1 |
| Cysteinesulfinic acid | 1680.7 | 10.655 | 252 | 16.7 | 3 | 1 |
| Maleamic acid | 1698.1 | 10.786 | 244 | 39.2 | 2 | 1 |
| L-Lysine (derivative not found) | 1704.0 | 10.830 | 84 | 9.7 | 3 | 1 |
| 2-aminoadipic acid | 1711.2 | 10.844 | 260 | 21.5 | 3 | 3 |
| Ribitol | 1712.4 | 10.893 | 217 | 20.4 | 5 | 1 |
| Glutamine | 1723.0 | 10.973 | 227 | 29.1 | 4 | 2 |
| Putrescine | 1740.9 | 11.107 | 174 | 17.3 | 4 | 1 |
| methyl 2,3,5-tris-O- Furanoside | 1743.0 | 11.123 | 217 | 37.0 | 3 | 3 |
| Glycerol-3-phosphate | 1751.2 | 11.184 | 357 | 18.5 | 4 | 1 |
| Ornithine | 1756.6 | 11.224 | 174 | 33.0 | 3 | 2 |
| Gluconic acid 1,4-lactone | 1758.9 | 11.242 | 217 | 23.6 | 4 | 1 |
| Glutamine | 1773.1 | 11.348 | 156 | 22.9 | 3 | 2 |
| Ethanolamine phosphate | 1778.1 | 11.391 | 299 | 8.4 | 4 | 1 |
| Methionine sulfoxide | 1782.9 | 11.422 | 128 | 5.1 | 3 | 1 |
| Phenylpyruvate | 1806.7 | 11.601 | 293 | 12.5 | 2 | 1 |
| Citric acid | 1808.0 | 11.610 | 273 | 18.4 | 4 | 1 |
| Ornithine | 1810.9 | 11.631 | 142 | 6.6 | 4 | 2 |
| Hypoxanthine | 1818.0 | 11.685 | 265 | 6.5 | 2 | 1 |
| Arginine (-NH_3_) | 1819.2 | 11.694 | 157 | 13.4 | 3 | 2 |
| Tagatose | 1822.7 | 11.720 | 217 | 12.5 | 5 | 3 |
| Allulose | 1833.0 | 11.797 | 217 | 26.5 | 5 (1 MeOx) | 3 |
| 1,5-anhydro-glucitol | 1840.3 | 11.855 | 204 | 17.0 | 5 | 1 |
| Dehydroascrobic acid (dimer) | 1844.5 | 11.884 | 316 | 25.1 | (2MeOx) | 1 |
| Tagatose-meto | 1843.6 | 11.877 | 173/157/147 | 32.1 | 5 | 3 |
| Fructose | 1851.3 | 11.935 | 307 | 13.9 | 5 (1 MeOx) | 1 |
| Lysine | 1853.2 | 11.949 | 174 | 30.2 | 3 | 2 |
| Sorbose | 1859.9 | 11.999 | 307 | 20.0 | 5 (1 MeOx) | 1 |
| Tyrosine | 1863.7 | 12.028 | 179 | 36.8 | 2 | 2 |
| 1-O-methyl-Galactopyranoside | 1865.5 | 12.041 | 204 | 22.5 | 4 | 1 |
| D-Glucopyranose | 1874.3 | 12.071 | 204 | 4.8 | 5 | 1 |
| Galactose | 1876.2 | 12.121 | 319 | 9.8 | 5 (1MeOx) | 3 |
| Adenine | 1877.5 | 12.131 | 264 | 20.4 | 2 | 1 |
| 3-(4-hydroxyphenyl)lactic acid | 1889.6 | 12.222 | 179 | 13.7 | 2 | 1 |
| Glucose | 1895.6 | 12.267 | 205 | 34.6 | 5 (1MeOx) | 1 |
| Lysine | 1908.8 | 12.356 | 156 | 8.3 | 4 | 2 |
| Histidine | 1915.1 | 12.396 | 154 | 43.8 | 3 | 2 |
| Tyrosine | 1932.5 | 12.506 | 280 | 17.8 | 3 | 2 |
| Ascorbic acid | 1934.7 | 12.520 | 332 | 12.5 | 4 | 1 |
| 2-Piperidinecarboxylic acid | 1937.4 | 12.538 | 84 | 38.9 | 1 | 1 |
| D-Glucopyranose | 1952.1 | 12.630 | 204 | 13.8 | 5 | 1 |
| Pantothenic acid | 1984.3 | 12.835 | 291 | 15.7 | 3 | 1 |
| 2-Methoxyestradiol | 1992.0 | 12.883 | 227 | 26.0 | 2 | 1 |
| Ribose-5-phosphate | 2008.7 | 12.989 | 315 | 29.0 | 5 | 3 |
| Inositol | 2011.4 | 13.006 | 318 | 10.3 | 6 | 3 |
| Xanthine | 2016.5 | 13.039 | 353 | 7.5 | 3 | 3 |
| Hexadecanoic acid (palmitic acid) | 2045.4 | 13.222 | 117 | 15.6 | 1 | 1 |
| Cysteinyl-glycine | 2049.9 | 13.251 | 220 | 33.4 | 3 | 1 |
| Dodecanedioic acid | 2063.7 | 13.338 | 117 | 8.6 | 2 | 1 |
| Myo-inositol | 2078.1 | 13.429 | 305 | 14.1 | 6 | 1 |
| Xylulose-5-phosphate | 2085.4 | 13.476 | 315 | 14.6 | 5 (1MeOx) | 1 |
| Uric acid | 2088.5 | 13.495 | 441 | 41.8 | 4 | 1 |
| Guanine | 2128.1 | 13.746 | 352 | 7.4 | 3 | 1 |
| trans-Caffeic acid | 2134.6 | 13.786 | 219 | 26.7 | 3 | 1 |
| Octadeca-1-ol | 2151.5 | 13.894 | 327 | 35.4 | 1 | 1 |
| 3-amino-propylphosphonate | 2176.6 | 14.054 | 424 | 33.5 | 3 | 3 |
| (9Z, 12Z)-9, 12-Octadecadienoic acid (linoleic acid) | 2210.8 | 14.259 | 75 | 28.9 | 1 | 1 |
| mannose-1-phosphoric acid | 2212.6 | 14.268 | 217 | 34.9 | 5 | 3 |
| 9-(E)-12-(Z)-Octadecadienoic acid | 2215.7 | 14.285 | 339 | 36.5 | 1 | 1 |
| Tryptophan | 2218.3 | 14.298 | 202 | 24.1 | 3 | 1 |
| Ribulose-5-phosphate | 2223.4 | 14.333 | 357 | 23.8 | 5 (1MeOx) | 3 |
| Stearic acid (octadecanoic acid) | 2241.9 | 14.422 | 117 | 12.0 | 1 | 1 |
| Hyodeoxycholic acid | 2270.4 | 14.598 | 579 | 40.2 | 3 | 3 |
| Fructose-1- or Fructose-6-phosphate | 2284.4 | 14.646 | 315 | 16.7 | 6 (1MeOx) | 3 |
| Cystine | 2287.2 | 14.661 | 218 | 24.5 | 4 | 3 |
| Mannose-6-phosphate | 2288.2 | 14.666 | 357 | 26.9 | 6 (1 MeOx) | 3 |
| Galactose-6-phosphate | 2300.2 | 14.729 | 359 | 34.6 | 6 (1 MeOx) | 3 |
| Oxalic acid, allyl tridecyl ester | 2343.4 | 14.952 | 315 | 23.4 | 0 | 3 |
| Glucose-6-phosphate | 2361.6 | 15.052 | 299 | 13.2 | 6 | 3 |
| Myo-Inositol-2-phosphate | 2407.5 | 15.294 | 318 | 10.8 | 7 (1 MeOx) | 1 |
| Pentadecan-1-ol | 2446.5 | 15.499 | 285 | 6.7 | 1 | 1 |
| Uridine | 2451.2 | 15.524 | 258 | 14.5 | 4 | 1 |
| Inosine | 2575.4 | 16.177 | 217 | 20.3 | 4 | 1 |
| Adenosine | 2639.2 | 16.513 | 230 | 34.7 | 3 | 3 |
| Cytidine | 2694.7 | 16.822 | 223 | 33.4 | 4 | 3 |
| Sucrose^#^ | 2715.5 | 16.926 | 361 | 9.1 | 8 | 1 |
| Guanosine | 2754.3 | 17.119 | 324 | 34.9 | 5 | 3 |
| Thymidine-5'-monophosphoric acid | 2817.6 | 17.443 | 81 | 16.9 | 3 | 3 |
| Uridine-5-monophosphate | 2830.5 | 17.528 | 169 | 22.6 | 5 | 1 |
| Inosine-5-phosphate derivative | 2877.3 | 17.729 | 169 | 28.0 | 5 | 3 |
| Adenosine-5-monophosphate | 3038.5 | 18.501 | 315 | 37.5 | 4 | 3 |
| Guanosine-5-monophosphate | 3050.2 | 18.557 | 169 | 24.2 | 4 | 3 |
| Cholesterol | 3191.8 | 19.193 | 329 | 18.6 | 1 | 1 |

Ⱶ R.I. and retention time values were averaged across all experiments for uninjected oocytes

† Quality Control (QC) relative S.D. = %

‡ ID indicates the criteria for correct identification: 1 = Metabolomexpress/AMDIS analysed and identified using the GOLM library based on Kovat’s Retention Index (RI) and quantification ion (m/z), confirmed using the GOLM Metabolome Database (2 = As for category 1 and confirmed using pre-made standard, 3 = NIST library reference match score > 700 and by manual matching of m/z peaks and RI data.

# All selection criteria and utilised databases give this metabolite a very strong match for the disaccharide sucrose and it is a high abundant metabolite in oocytes. However, sucrose has never been convincingly detected in animal tissue and animal genomes lack any recognisable enzyme for the synthesis of the D-fructose-D-glucose glycosidic bond.

**Table S5: Metabolites isolated from *X. laevis* oocytes by LC-MS/MS aqueous phase extraction**

| **Metabolite name** | **Average R.T. (min)** | **Average m/z ion** | **QC Relative S.D.^†^ (%)** |
| --- | --- | --- | --- |
| 5-Methylcytosine | 3.79 | 126.0661 | 7.1485 |
| Acetylcarnitine | 2.36 | 204.1225 | 4.24866 |
| Acetylcholine | 4.1 | 146.1171 | 37.23456 |
| Adenine | 2.52 | 136.0617 | 13.29448 |
| L-Alanine | 1.74 | 90.0553 | 15.04745 |
| D-2-Aminoadipic acid | 5.35 | 162.0759 | 3.33546 |
| L-Arginine | 8.58 | 175.1186 | 12.30874 |
| Asparagine | 5.64 | 133.0606 | 1.06083 |
| Aspartic acid | 6.24 | 134.0445 | 11.95573 |
| Betaine | 3.57 | 118.0862 | 6.95487 |
| Biotin | 1.44 | 245.0949 | 8.78669 |
| Choline | 3.14 | 104.1071 | 1.21395 |
| Citrulline | 5.89 | 176.1028 | 3.88737 |
| Creatinine | 3.8 | 114.0662 | 7.5295 |
| Cysteine | 4.69 | 122.027 | 11.2624 |
| L-Glutamic acid | 5.76 | 148.0601 | 3.57723 |
| Glutamine | 5.53 | 147.0762 | 21.87283 |
| Glutathione (reduced) | 5.56 | 308.0904 | 5.98074 |
| Glycine | 5.71 | 76.0397 | 6.07182 |
| Guanine | 3.34 | 152.0564 | 1.77192 |
| Guanine | 3.33 | 303.1055 | 9.52584 |
| L-Histidine | 8.59 | 156.0765 | 7.04742 |
| Isoleucine | 3.45 | 132.1017 | 2.64271 |
| Leucine | 3.23 | 132.1013 | 7.52253 |
| L-lysine | 8.47 | 147.1125 | 9.03476 |
| L-Methionine | 3.71 | 150.0582 | 8.21433 |
| Pantothenic acid | 1.74 | 220.1174 | 11.22185 |
| Phenylalanine | 3.04 | 166.086 | 5.25743 |
| Phosphocreatine | 6.55 | 212.0426 | 9.38011 |
| DL-Pipecolinic acid | 6.98 | 130.0862 | 24.07875 |
| Proline | 4.24 | 116.0706 | 16.10093 |
| Serine | 5.96 | 106.05 | 3.05111 |
| Maltose | 5.5 | 343.1226 | 9.6986 |
| Taurine | 5.14 | 126.0219 | 7.24399 |
| Threonine | 5.36 | 120.0656 | 3.48497 |
| Tryptophan | 3.32 | 205.0968 | 6.48839 |
| Tyrosine | 4.53 | 182.0809 | 8.01648 |
| Valine | 3.52 | 118.0862 | 6.95484 |

† Quality Control (QC) relative S.D. = %

**Table S6: Homologous mTORC1 pathway components in humans and *Xenopus laevis***

| **Role in mTORC1 Pathway** | **Protein** | **Human Sequence (UniProt)** | ***X.laevis* Sequence (UniProt)** |
| --- | --- | --- | --- |
| mTORC1 complex and lysosomal signalling | mTOR | P42345 | A0A1L8FKW5 |
|  | Raptor | Q8N122 | A0A1L8ETG8 |
|  | Rag A | Q7L523 | Q6IP38^°^ |
|  | Rag B | Q5VZM2 |  |
|  | Rag C | Q9HB90 | A0A1L8G360 |
|  | Rag D | Q9NQL2 | A0A1L8G996 |
|  | Rheb GTPase | Q15382 | A0A1L8FQH6 |
|  | V-type proton ATPase^†^ | Q9Y487 | A0A1L8EKJ7 |
|  | Ragulator LAMTOR 1 | Q6IAA8 | A0A1L8HBN4 |
|  | Ragulator LAMTOR 2 | Q9Y2Q5 | A0A1L8F4K6 |
|  | Ragulator LAMTOR 3 | Q9UHA4 | A0A1L8GAW7 |
|  | Ragulator LAMTOR 4 | Q0VGL1 | Not found^‡^ |
|  | Ragulator LAMTOR 5 | O43504 | A0A1L8H702 |
|  | SLC38A9 | Q8NBW4 | A0A1L8HRK2 |
|  | FLCN complex – folliculin | Q8NFG4 | A0A1L8EQZ5 |
|  | FLCN complex – FNIP1 | Q8TF40 | A0A1L8GVG0 |
|  | FLCN complex – FNIP2 | Q9P278 | A0A1L8HU87 |
| Upstream amino acid signalling components | CASTOR 1 | Q8WTX7 | A0A1L8HZR1 |
|  | ADP ribosylation factor1 (Arf-1) | P84077 | A0A1L8FQF3 |
|  | CASTOR 2 | A6NHX0 | A0A1L8HG91 |
|  | Sestrin 1 | Q9Y6P5 | A0A1L8G963 |
|  | Sestrin 2 | P58004 | A0A1L8G2X6 |
|  | **GATOR2 complex – MIOS** | Q9NXC5 | A0A1L8FR11 |
|  | **GATOR2 complex – WDR59** | Q6PJI9 | A0A1L8GLG4 |
|  | **GATOR2 complex – SEH1** | Q96EE3 | A0A1L8FYF0 |
|  | **GATOR2 complex – SEC13** | P55735 | Q7ZYJ8 |
|  | **GATOR1 complex – DEPDC5** | O75140 | A0A1L8I0B3 |
|  | **GATOR1 complex – NPRL2** | Q8WTW4 | A0A1L8GHF2 |
|  | **GATOR1 complex – NPRL3** | Q12980 | A0A1L8EXD1 |
|  | SAMTOR | Q1RMZ1 | A0A1L8GYV9 |
| Downstream effectors of mTORC1 | S6K1 (isoform α1) | Q15418 | Q6GR32 |
|  | S6K1 (isoform β1) | P23443 | A0A1L8HFU6 |
|  | 4E-BP1 | Q13541 | Q6DJI1 |
|  | S6 (40S ribosomal protein S6) | P62753 | A0A1L8HNW4 |

**°** *X.laevis* has only 3 homologs of Ras-related GTPases (Rag), with human Rag A and B showing greatest sequence conservation with the same Xenopus protein.

**†** Lysosomal V-type proton ATPase 116 kDa subunit A isoform 2. All other human V-type ATPase subunits have single, highly conserved homologues in *X.laevis*.

‡ *Xenopus tropocalis*, the nearest sequenced relative of *X.laevis*, has a LAMTOR 4 homologue, making this finding significant

**References**

Bröer, A., Brookes, N., Ganapathy, V., Dimmer, K.S., Wagner, C.A., Lang, F., et al. (1999). The astroglial ASCT2 amino acid transporter as a mediator of glutamine efflux. *J Neurochem* 73(5)**,** 2184-2194.

Broer, A., Rahimi, F., and Broer, S. (2016). Deletion of Amino Acid Transporter ASCT2 (SLC1A5) Reveals an Essential Role for Transporters SNAT1 (SLC38A1) and SNAT2 (SLC38A2) to Sustain Glutaminolysis in Cancer Cells. *J Biol Chem* 291(25)**,** 13194-13205. doi: 10.1074/jbc.M115.700534.

Chubb, S., Kingsland, A.L., Broer, A., and Broer, S. (2006). Mutation of the 4F2 heavy-chain carboxy terminus causes y+ LAT2 light-chain dysfunction. *Mol Membr Biol* 23(3)**,** 255-267. doi: J31772135X6855M1 [pii] 10.1080/09687860600652968 [doi].

Seow, H., Broer, S., Broer, A., Bailey, C., Potter, S., Cavanaugh, J., et al. (2004). Hartnup disorder is caused by mutations in the gene encoding the neutral amino acid transporter SLC6A19. *Nature Genetics* 36(9)**,** 1003-1007. doi: 10.1038/ng1406.

Wagner, C.A., Broer, A., Albers, A., Gamper, N., Lang, F., and Broer, S. (2000). The heterodimeric amino acid transporter 4F2hc/LAT1 is associated in Xenopus oocytes with a non-selective cation channel that is regulated by the serine/threonine kinase sgk-1. *J Physiol (Lond)* 526(Pt 1)**,** 35-46.
